# Multimodal classification of molecular subtypes in pediatric acute lymphoblastic leukemia

**DOI:** 10.1038/s41698-023-00479-5

**Published:** 2023-12-08

**Authors:** Olga Krali, Yanara Marincevic-Zuniga, Gustav Arvidsson, Anna Pia Enblad, Anders Lundmark, Shumaila Sayyab, Vasilios Zachariadis, Merja Heinäniemi, Janne Suhonen, Laura Oksa, Kaisa Vepsäläinen, Ingegerd Öfverholm, Gisela Barbany, Ann Nordgren, Henrik Lilljebjörn, Thoas Fioretos, Hans O. Madsen, Hanne Vibeke Marquart, Trond Flaegstad, Erik Forestier, Ólafur G. Jónsson, Jukka Kanerva, Olli Lohi, Ulrika Norén-Nyström, Kjeld Schmiegelow, Arja Harila, Mats Heyman, Gudmar Lönnerholm, Ann-Christine Syvänen, Jessica Nordlund

**Affiliations:** 1 Department of Medical Sciences, Molecular Precision Medicine and Science for Life Laboratory, Uppsala University, Uppsala, Sweden; 2 Department of Women’s and Children’s Health, Uppsala University, Uppsala, Sweden; 3 Department of Oncology-Pathology, Karolinska Institutet, Stockholm, Sweden; 4 Institute of Biomedicine, School of Medicine, University of Eastern Finland, Kuopio, Finland; 5 Tampere Center for Child, Adolescent and Maternal Health Research, Faculty of Medicine and Health Technology, Tampere University, Tampere, Finland; 6 Tampere University Hospital, Tays Cancer Center, Tampere, Finland; 7 Department of Pediatrics, Kuopio University Hospital, Kuopio, Finland; 8 Department of Molecular Medicine and Surgery and Center for Molecular Medicine, Karolinska Institutet, Stockholm, Sweden; 9 Department of Clinical Genetics, Karolinska University Hospital, Stockholm, Sweden; 10 Division of Clinical Genetics, Dept. of Laboratory Medicine, Lund University, Lund, Sweden; 11 Department of Clinical Immunology, Copenhagen University Hospital Rigshospitalet, Copenhagen, Denmark; 12 Department of Clinical Medicine, Faculty of Health and Medical Sciences, University of Copenhagen, Copenhagen, Denmark; 13 Department of Pediatrics, Tromsø University and University Hospital, Tromsø, Norway; 14 For the Nordic Society of Pediatric Hematology and Oncology (NOPHO), Stockholm, Sweden; 15 Department of Medical Biosciences, University of Umeå, Umeå, Sweden; 16 Pediatric Hematology-Oncology, Children’s Hospital, Barnaspitali Hringsins, Landspitali University Hospital, Reykjavik, Iceland; 17 New Children’s Hospital, Helsinki University Central Hospital and University of Helsinki, Helsinki, Finland; 18 Department of Clinical Sciences, Pediatrics, Umeå University, Umeå, Sweden; 19 Pediatrics and Adolescent Medicine, Rigshospitalet, and the Medical Faculty, Institute of Clinical Medicine, University of Copenhagen, Copenhagen, Denmark; 20 Childhood Cancer Research Unit, Karolinska Institutet, Astrid Lindgren Children’s Hospital, Karolinska University Hospital, Stockholm, Sweden

**Keywords:** Molecular medicine, Acute lymphocytic leukaemia

## Abstract

Genomic analyses have redefined the molecular subgrouping of pediatric acute lymphoblastic leukemia (ALL). Molecular subgroups guide risk-stratification and targeted therapies, but outcomes of recently identified subtypes are often unclear, owing to limited cases with comprehensive profiling and cross-protocol studies. We developed a machine learning tool (ALLIUM) for the molecular subclassification of ALL in retrospective cohorts as well as for up-front diagnostics. ALLIUM uses DNA methylation and gene expression data from 1131 Nordic ALL patients to predict 17 ALL subtypes with high accuracy. ALLIUM was used to revise and verify the molecular subtype of 281 B-cell precursor ALL (BCP-ALL) cases with previously undefined molecular phenotype, resulting in a single revised subtype for 81.5% of these cases. Our study shows the power of combining DNA methylation and gene expression data for resolving ALL subtypes and provides a comprehensive population-based retrospective cohort study of molecular subtype frequencies in the Nordic countries.

## Introduction

Pediatric acute lymphoblastic leukemia (ALL) comprises a heterogeneous group of patients who can be stratified into subgroups based on the presence of recurrent cytogenetic aberrations, which are important predictors of clinical outcome^1,2^. Subtypes of pediatric B-cell precursor ALL (BCP-ALL) are often characterized by large-scale chromosomal aberrations, including abnormal chromosomal numbers, translocations that give rise to expressed fusion genes, or other structural rearrangements. Before next-generation sequencing (NGS)-based methods were introduced into clinical practice, as many as 30% of all BCP-ALL cases either lacked conclusive results from standard cytogenetic analyses (denoted undefined) or were negative for the subtype-defining aberrations (denoted B-other) and therefore subtype information was not available for treatment stratification or disease monitoring in this large group of patients^3^. Recent application of high-resolution transcriptome sequencing (RNA-seq) has enabled the discovery of new oncogenic subgroups characterized by fusion genes, such as *DUX4* (*DUX4*-r), *ZNF384* (*ZNF384*-r), *MEF2D* (*MEF2D*-r) and *NUTM1* (*NUTM1*-r) rearrangements^4–15^, as well as subtype-like signatures, such as *BCR*::*ABL1*-like/“Ph-like”^16,17^ or *ETV6*::*RUNX1*-like/”ER-like”^7,18^, and the PAX5-driven subtypes, PAX5 alteration (PAX5alt) and PAX5 P80R^19–22^. The clinical significance of the recently identified subtypes is often unclear, owing to the limited number of cases and differences between protocols and studies^7,8,20,23,24^. Therefore, retrospective ALL cohort analyses have been particularly powerful for studying rare ALL subtypes due to the large sample sizes available in biobanks and the prolonged period of follow-up to collect sufficient data on rare events.

Most of the recurrent molecular alterations in ALL are strongly associated with gene expression (GEX) profiles^24,25^. RNA-seq has since emerged as a powerful tool for the identification of both fusion genes and GEX subtype profiling in a single assay^26,27^, which promises to replace cumbersome standard karyotyping (G-banding), PCR-based and fluorescence in situ hybridization (FISH)-based methods in a clinical diagnostic setting^28^. Compared to DNA, RNA is prone to degradation, making it challenging to obtain high-quality RNA for retrospective cohort analyses. However, epigenetic profiling of DNA methylation (DNAm) using arrays or next-generation sequencing (NGS) has demonstrated comparable subtype-specific distributions in ALL cells^29–32^. DNAm is advantageous as an analyte due to its ability to identify methylation patterns associated with disease in degraded archival samples^33^. Leveraging biobank samples and retrospective cohort studies can provide valuable insights into long-term disease outcomes that may be challenging to obtain through prospective study designs, particularly for rare ALL subtypes.

**Fig. 1:**
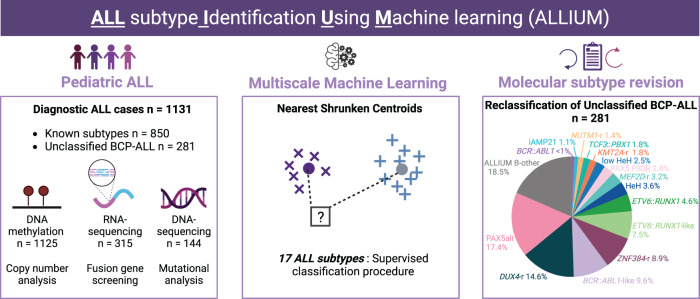
Study overview. DNA methylation (DNAm, 450k arrays), gene expression (GEX, RNA-sequencing), and somatic mutation (WGS, targeted sequencing) data were generated from 1131 patients treated on the Nordic Society for Pediatric Hematology and Oncology (NOPHO) protocols diagnosed between 1996 and 2013. In total, the subtype of 281 of the BCP-ALL patients (24.8% of the entire ALL cohort) was unclassified at diagnosis. Molecular screening was performed based on a combination of cytogenetics, fusion gene screening, mutational analysis, and copy number analysis. Molecular screening resolved the subtype of 127 BCP-ALL patients. The remaining 154 patients were denoted “B-other”. A supervised classification method (ALLIUM) was used to build subtype-specific models based on two modalities (DNAm and GEX) for 17 of the known molecular subtypes of ALL. ALLIUM re-classified the subtype of 102 B-other patients. This study expanded the scope of known subtypes across the entire cohort resulting in 1079 with known subtype (95.4% of the entire ALL cohort). The 52 patients remaining unclassified at the end of the study are referred to as ALLIUM B-other.

In the present investigation, we describe a multimodal machine learning classification tool, ALL subtype Identification Using Machine learning (ALLIUM) that uses DNAm and/or GEX signatures (Fig. 1). We trained and applied ALLIUM to a large cohort of 1131 Nordic patient samples and determined the frequencies of recent genetic subtypes, which led to the revision of molecular subtypes in 81.5% of the unclassified BCP-ALL cases.

## Results

### Molecular characteristics and data generation

Diagnostic bone marrow aspirates or peripheral blood samples from 1131 Nordic ALL patients (*n* = 1025 BCP-ALL and *n* = 106 T-ALL) were obtained from a population based cohort diagnosed between 1996 and 2013, and enrolled in the Nordic Society of Pediatric Hematology and Oncology (NOPHO) −92, −2000, −2008, EsPh-ALL, or Interfant treatment protocols^34–37^. Genome-wide CpG methylation levels were analyzed in 1125 DNA samples (1125 patients) using 450k arrays (DNAm dataset) and RNA-sequencing was performed in 328 RNA samples (315 patients, GEX dataset). Molecular subtypes were assigned based on standard cytogenetic analysis at ALL diagnosis^38^, where a total of 850 patients (75%) had an established molecular subtype and 281 were denoted unclassified BPC-ALL (Supplementary Data 1). We initially screened our cohort for the molecular ALL subtypes outlined by the International Consensus Classification (ICC)^2^ using a combination of genome-wide CNA detection, fusion gene screening (Supplementary Fig. 1), and targeted mutational assessment for *PAX5* p.Pro80Arg, *IKZF1* p.Asn159Tyr, and *ZEB2* p.His1038Arg (Supplementary Data 2). This analysis, combined with putative revised molecular subtype information from previously published results^7,9,30,39–41^, identified 127 patients from the unclassified BCP-ALL group (45.2%) who belonged to one of the ICC subtypes. In total, this yielded 977 ICC subtype-defined cases (Table 1). Of note, this included 27 patients with established subtypes missed by routine diagnostics: HeH (*n* = 9), *ETV6*::*RUNX1* (*n* = 9), *KMT2A*-r (*n* = 4), *TCF3*::*PBX1* (*n* = 3), *BCR*::*ABL1* (*n* = 1), and iAMP21 (*n* = 1). One patient (ALL_913) was re-labelled from HeH to *DUX4*-r, after confirmation of the presence of the *IGH-DUX4* fusion gene and a modal number of 46 chromosomes. The 30 patients with dic(9;20) aberrations^42^ were re-labelled as PAX5alt. The remaining 154 cases were denoted as B-other after the application of the aforementioned genomic techniques failed to resolve their subtype (Table 1).

**Table 1:** Overview of the 1131 ALL patients by ICC subtype prior to multimodal classification.

Molecular subtype*	# Patients DNAm	# Patients GEX	# Patients Total	Age (sd)	# Male/Female
Total patients	1125	315	1131	6.35 ( ± 4.39)	615/516
B-other	154	67	154	8.48 ( ± 4.98)	83/71
ICC subtype-defined	971	248	977	6.01 ( ± 4.19)	532/445
HeH	309	46	310	5.11 ( ± 3.51)	166/144
low HeH	5	3	5	3.27 ( ± 1.33)	2/3
iAMP21	20	16	21	10.23 ( ± 3.95)	13/8
Hypodiploidy	10	0	10	9.52 ( ± 4.58)	6/4
*ETV6*::*RUNX1*	274	32	275	4.93 ( ± 2.55)	144/131
*ETV6*::*RUNX1*-like	12	10	12	4.13 ( ± 3.66)	5/7
*KMT2A*-r	61	14	62	2.76 ( ± 4.22)	26/36
*NUTM1-*r	3	3	3	9.46 ( ± 7.78)	1/2
PAX5alt	49	30	50	5.57 ( ± 4.94)	23/27
PAX5 P80R	5	4	5	12.35 ( ± 5.83)	4/1
*TCF3*::*PBX1*	37	10	37	8.07 ( ± 4.55)	17/20
*MEF2D-*r	9	8	9	11.3 ( ± 4.57)	3/6
*BCR*::*ABL1*	25	10	25	8.92 ( ± 3.82)	15/10
*BCR*::*ABL1*-like	10	7	10	8.82 ( ± 5.75)	8/2
*DUX4-*r	20	19	20	9.72 ( ± 3.11)	11/9
*ZNF384-*r	17	17	17	9.02 ( ± 3.78)	10/7
T-ALL	105	19	106	9.08 ( ± 4.61)	78/28

^*^Molecular subtypes were labelled according to the International Consensus Classification (ICC). Fluorescence in situ hybridization and/or reverse-transcriptase polymerase chain reaction were applied at ALL diagnosis to identify established subtypes *ETV6*::*RUNX1*, *TCF3*::*PBX1*, *KMT2A*-r, dic(9;20), iAMP21. High hyperdiploidy (HeH) was defined as a modal number ≥ 51 chromosomes or DNA Index (DI) 1.12–1.35. Hypodiploidy was defined as < 40 chromosomes and included low-hypodiploidy with 30–39 chromosomes or DI 0.6-0.84 and near-haploidy (NH) with 24–29 chromosomes or DI < 0.6. Low HeH was defined as 47–50 chromosomes as determined by array-based copy number analysis and lack of other subtype-defining aberrations. All karyotypes were centrally reviewed. The subtypes of previously unclassified BCP-ALL cases were revised using a combination of genome-wide CNA detection, fusion gene screening and targeted mutational assessment (Supplementary Data 2).

### ALLIUM is a highly sensitive method for molecular ALL subtype classification

In order to design a DNAm and GEX-based classifiers for ALL, the 977 patients with known ICC molecular subtypes defined based on updated molecular analysis were split into design and hold-out datasets to create and validate the ALLIUM classifier (Table 2). An internally produced replication set (*n* = 13, GEX) and three external datasets (GEX: GSE161501^43^, GEX: GSE228632 and DNAm: GSE56600^31^) were used for additional independent subtype verification. ALLIUM is based on nearest shrunken centroid (NSC)^44^ models consisting of DNAm and GEX data in a one vs. rest approach. Subtypes with similar molecular profiles, i.e. those characterized by aneuploidies (HeH, low HeH, iAMP21, hypodiploidy), *ETV6* gene rearrangements (*ETV6*::*RUNX1*, *ETV6*::*RUNX1*-like), and the Philadelphia (ph) chromosome (*BCR*::*ABL1*, *BCR*::*ABL1*-like) were handled in a different manner. For these subtypes, a two-step procedure with initial classification on the group level, followed by a one-vs-one or a multi-class classification within the group was applied (Supplementary Materials and Methods and Supplementary Fig. 2–3). Moreover, to identify misclassification errors due to low blast count, control classifiers for DNAm and RNA were built utilizing data available from ALL patients in remission or healthy blood donors^45^. As the output contained probability scores for each classifier, multiple subtype classifications could occur. Therefore, we proceeded with a multi- to single-class transformation, assigning the subtype with the highest probability score for each sample.

**Table 2:** Classifier performance and concordance.

	Dataset	No of samples	sensitivity	specificity	Concordance with true ALL subtype *n* (%)
DNAm GSE49031 and 10.17044/scilifelab.22303531	Design	819	0.883	0.999	759 (92.7)
	Hold-out	152	0.834	0.998	136 (89.5)
	Discovery (B-other)	154	–	–	–
DNAm GSE56600	Validation	133	0.872	0.992	112 (84.2)
	B-other/unknown	94	–	–	–
GEX GSE227832	Design	207	0.964	0.998	199 (96.1)
	Hold-out	41	0.953	0.999	38 (92.7)
	Replication	12	0.792	1.000	10 (83.3)
	Replication (B-other)	1	–	–	–
	Discovery (B-other)	67	–	–	–
GEX GSE228632	Validation	55	0.974	0.999	53 (96.4)
	B-other/unknown	10	–	–	–
GEX GSE161501	Validation	19	1.000	1.000	19 (100)

**Fig. 2:**
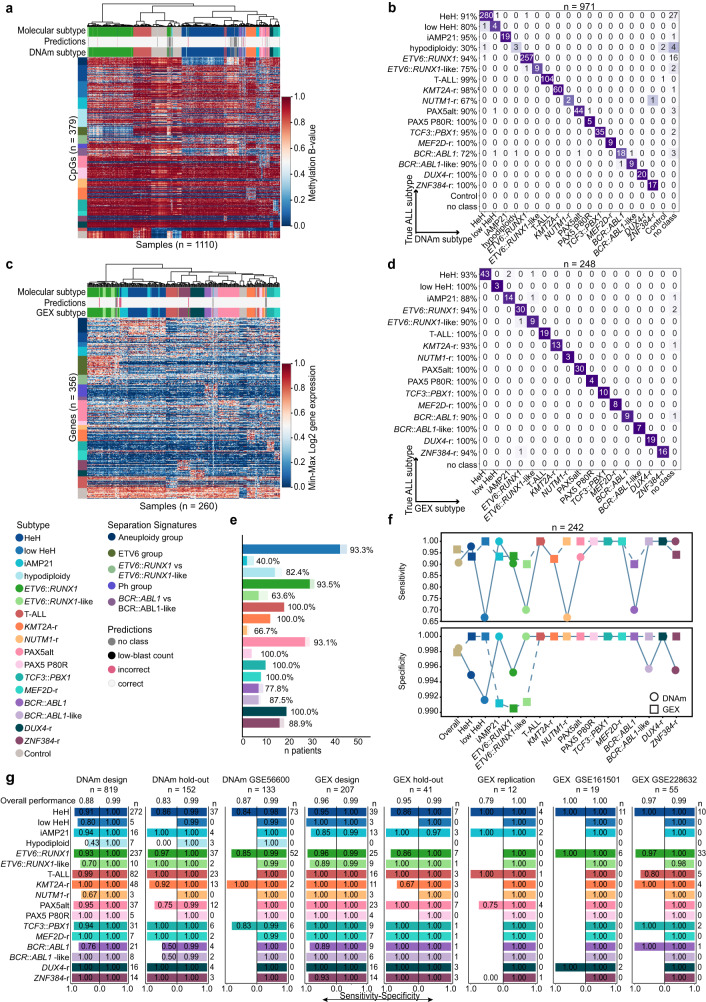
Evaluation of model performance. **a** Unsupervised hierarchical clustering based on the DNA methylation (DNAm) β-values of 379 CpG sites across molecularly defined patients (*n* = 971) and control samples (*n* = 139). The heatmap shows the DNA methylation β-value for each CpG (y-axis) and sample (x-axis). The color key is indicated to the right of the panel. **b** Confusion matrix showing the concordance between ALLIUM DNAm subtype predictions (x-axis) and true molecular subtypes (y-axis) for 971 patients. The numbers indicate the number of patients by subtype. **c** Unsupervised hierarchical clustering based on gene expression (GEX) levels of 356 genes across molecularly defined patients (*n* = 248) and control samples (*n* = 12). The heatmap shows the min-max scaled log2 gene expression levels of the 356 genes (y-axis) by sample (x-axis). The color key for the heatmap is indicated in the right side of the panel. **d** Concordance between ALLIUM GEX subtype predictions (x-axis) and true molecular subtypes (y-axis) for 248 patients analyzed with ALLIUM GEX. **e** Barplots showing the degree of concordance between ALLIUM DNAm and GEX predictions for 242 samples with data from both modalities. The subtype is indicated along the y-axis and the number of patients along the x-axis. The light bars represent the overall number of predictions per subtype and the darker bars indicate the number predictions concordant between DNAm and GEX. Patients with “no class” predictions (*n* = 9) are not shown. **f** Line plots demonstrating the sensitivity (top) and specificity (bottom) of ALLIUM DNAm (circle) and GEX (square) models overall and by subtype for the 242 patients analyzed with both data modalities. **g** Bi-directional barplots showing the sensitivity and specificity by subtype for the design, hold-out, replication, DNAm GSE56600, GEX GSE161501 and GEX GSE228632 datasets. The sensitivity is indicated by the left-sided bar, while the specificity is indicated by the right-sided bar for each dataset and subtype. The overall performance is shown on the top of each barplot. The number of patients in each dataset by subtype is indicated to the right of each barplot.

ALLIUM identified 379 CpGs and 356 genes as most informative for subtype determination (Supplementary Data 3–4). Unsupervised analysis of samples with known subtype revealed clear subtype-driven clustering (Fig. 2a–c, Supplementary Fig. 4). We evaluated the models using hold-out, replication and independent external validation datasets (Table 2, Supplementary Data 5–14). The classifiers were highly predictive overall, with 87.0% concordance between DNAm and true molecular subtype and 94.5% overall concordance between GEX and true molecular subtype (Fig. 2b–d). Both ALLIUM modalities achieved high specificity (>0.99) across the datasets, but the DNAm classifer displayed lower sensitivity (range 0.83 - 0.88) than the GEX classifier (range 0.79–1.00) (Table 2). Both DNAm and GEX classification was performed in samples from 242 patients in our dataset, enabling us to directly compare the two models (Fig. 2e, f). ALLIUM GEX resulted in 95.9% overall concordance with true subtype, 0.96 sensitivity and 0.99 specificity, while ALLIUM DNAm resulted in 93.8% overall concordance, 0.91 sensitivity and 0.99 specificity across these 242 patients (Fig. 2f, Supplementary Data 15). Only 19 cases showed discrepant result between the GEX and/or DNAm classifiers and true subtype. The GEX classifier was more often correct (*n* = 9), while the DNAm was correct in four cases. A complete mismatch was observed for the remaining six cases, including four cases with no class assignment (Supplementary Data 16). Of the nine patients that were correctly predicted by GEX, the DNAm classifier was unable to return a prediction for five cases (no-class), which supports our previous observation that ALLIUM DNAm is less sensitive than the GEX classifier (Supplementary Data 16). To summarize, the performance of the GEX and DNAm classifiers was largely consistent across the design, hold-out, replication and external validation datasets across the subtypes (Fig. 2g, Supplementary Fig. 4, Supplementary Data 14).

### Functional annotation of genes and CpG sites identified by ALLIUM

**Fig. 3:**
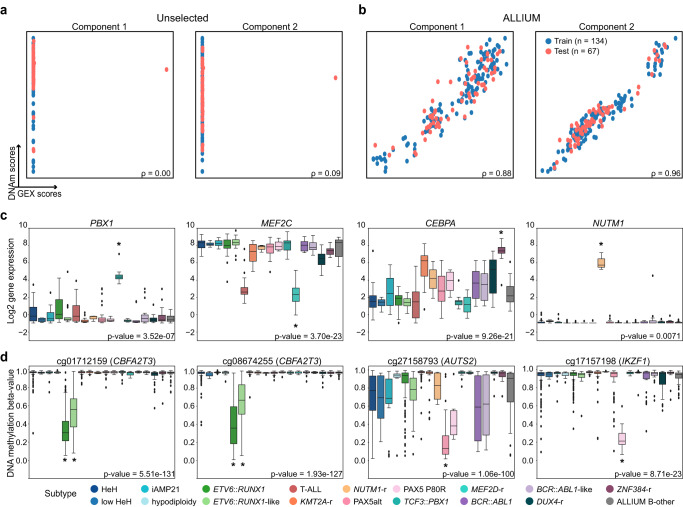
Subtype-specific signatures determined by ALLIUM. Cross-decomposition analysis with Partial Least Squares (PLS) Canonical analysis. The UMAP plots indicate components 1 and 2 for **a** the DNAm (*n* = 167,353) vs the GEX (*n* = 19,774) unselected signatures, and **b** the ALLIUM DNAm (*n* = 379) and GEX (*n* = 356) signatures (right). The points indicate the training (67%, blue) and test sets (33%, red). The Pearson’s correlation coefficient for the comparing modalities per component is denoted in the title of each plot. Boxplots demonstrating the **c** GEX levels for four selected genes across 315 patients grouped by revised molecular subtype. **d** DNAm levels for four selected CpG sites across 1125 patients by revised molecular subtype. The boxes are color-coded by respective subtype according to the key at the bottom of the panel. The Benjamini-Hochberg (BH) corrected Kruskal-Wallis H-test *p* value indicates the statistical significance between subtypes (bottom right). Asterisks indicate the subtype(s) for which ALLIUM chose each specific CpG or GEX signature. The lines (whiskers) on the boxplots represent the distribution of residual data points beyond the lower and upper quartiles.

Next, we explored the relationship between the features (CpG sites and genes) identified by ALLIUM. Initially, we assessed the covariance between the datasets using cross-decomposition analysis, focusing patients in the design cohort with overlapping DNAm and GEX data (*n* = 201). We analyzed all the available features, totaling 19,774 genes and 167,353 CpG sites, as well as the 356 genes and 379 CpG sites selected by ALLIUM. Notably, the features chosen by ALLIUM exhibited substantial correlation for components 1 (88%) and 2 (96%), while the correlation among all features was consistently below 10% (Fig. 3a, b). The strong correlation highlights a potentially biologically relevant association between the features selected by the two ALLIUM modalities.

To further explore this association, we considered the genomic distribution, the extent and magnitude of gene expression and DNA methylation changes, as well as their regulation across the features specific for the different subtypes. The features were distributed across all chromosomes, with no significant overall enrichment in the genomic locations of CpG sites or genes (FDR *q* value > 0.05, Supplementary Fig. 5 and Supplementary Data 17–18). However, the CpGs sites did show a significant enrichment in intergenic “open sea” regions outside of CpG islands (FDR *q* value < 0.0001, Supplementary Data 19).

We assessed the directionality of the subtype-defining features, and observed they were more frequently hypomethylated at the CpG level (Supplementary Fig. 6) and displayed greater expression levels and variability at the gene expression level (Supplementary Fig. 7). Further investigation of the genomic overlap between CpG sites and genes revealed 21 CpG sites that overlapped with the genomic location of 18 ALLIUM GEX genes (Supplementary Data 20–21). Among these, eight CpG sites located in seven genes were selected for the same subtype. For example, PAX5alt shared four CpG sites in the *RAPGEF4*, *CALN1* and *NAV2* genes, the aneuploidy group had one CpG site located within each of the *LCN6* and *CELSR1* genes, the *ETV6* group had one CpG site in *FARP1*, and *TCF3*::*PBX1* had one CpG site in *KANK1*. For these genes we observed an inverse relationship between methylation level and gene expression (Pearson’s correlation coefficient = −0.58, Supplementary Fig. 8).

Importantly, ALLIUM consistently selected well-known ALL genes such as *NUTM1* for *NUTM1*-r, *PBX1* for *TCF3*::*PBX1*, *MEF2C* for *MEF2D*-r, *CEBPA* for *ZNF384*-r, CpG sites in *CBFA2T3* for the *ETV6*-group, the expression of *CDKN2A* and CpG sites in *AUTS2* for the PAX5alt group, along with CpG sites in *ETV6, RUNX2*, and *IKZF1* for PAX5 P80R (Fig. 3c, d). These findings cumulatively underscore that ALLIUM classifiers consistently selected biologically relevant features for characterizing the subtypes.

### Comparisons of model performance

**Fig. 4:**
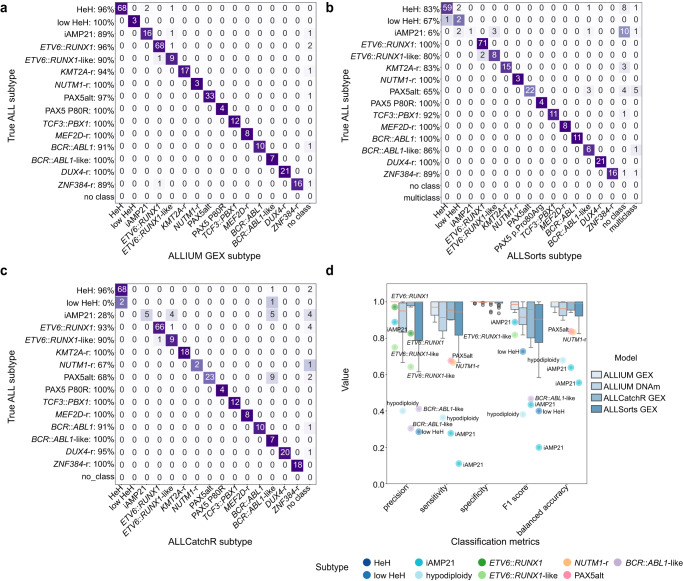
Performance of ALLIUM, ALLSorts and ALLCatchR. **a** Concordance between ALLIUM GEX subtype predictions (x-axis) and true molecular subtypes (y-axis) for 309 BCP-ALL samples of known subtype (95.5%, 295/309). **b** Concordance between ALLSorts subtype predictions (x-axis) and true molecular subtype (y-axis) (83.5%, 258/309). **c** Concordance between ALLCatchR subtype predictions (x-axis) and true molecular subtype (y-axis) (87.4%, 270/309). ALLCatchR was not trained on low HeH. **d** Boxplots demonstrating classification performance, including precision, sensitivity, specificity, F1 score and accuracy (balanced) for the three GEX models (*n* = 309 samples) and ALLIUM DNAm (*n* = 1104 samples with known subtype). The lines (whiskers) on the boxplots represent the distribution of residual data points beyond the lower and upper quartiles.

Several GEX-based methods for ALL subtyping have been developed independently. We evaluated the ALLIUM GEX classifier against ALLSorts^26^ and ALLCatchR^27^. As both classifiers are trained specifically for BCP-ALL, we removed T-ALL from the comparison. ALLIUM GEX, ALLSorts, and ALLCatchR were evaluated for the 309 BCP-ALL samples of known subtype across all the five GEX datasets included herein. Overall, the three classifiers performed similarly (Fig. 4a–c), although notable differences between ALLIUM GEX, ALLSorts, and ALLCatchR, respectively, were observed for classification of PAX5alt (100%, 71%, 71%), HeH (86%, 71%, 86%), and iAMP21 (100%, 33%, 67%) using our hold-out dataset (Supplementary Figs. 4 and 9–12). Specifically, we noted that ALLSorts and ALLCatchR predicted three (including a multi-class case) and eight out of 14 PAX5alt patients with dic(9;20) as *BCR*::*ABL1*-like, respectively. ALLIUM was not trained on *BCL2/MYC*, *IKZF1 N159Y*, *HLF*, *CEB* and *CDX2*::*UBTF* subtypes and ALLSorts and ALLCatchR did not predict any of these rare subtypes in our data sets.

Overall, ALLIUM DNAm performed similarly to the GEX models (Fig. 4d). No other model is currently available for subtyping in ALL by DNA methylation, with the exception of a model built by us previously for eight ALL subtypes^30^. We compared the 379 CpG sites selected by ALLIUM to our previous classifier (*n* = 232 CpG sites), which resulted in 28.9% (67/232) overlapping sites with ALLIUM (Supplementary Data 22).

### Resolved molecular subtypes of unclassified BCP-ALL

**Fig. 5:**
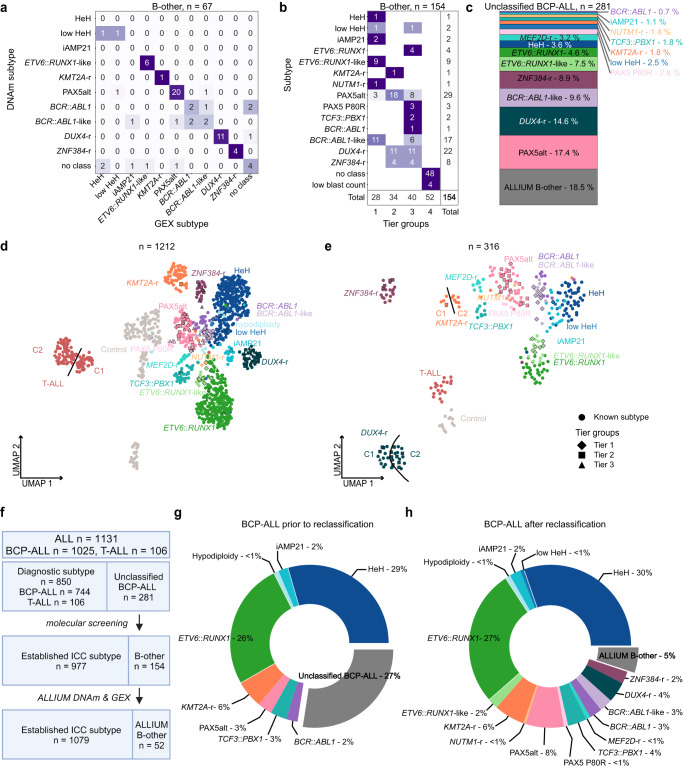
Frequencies of molecular subtypes. **a** Concordance of ALLIUM subtype estimation for 67 B-other patients with both DNA methylation (DNAm, x-axis) and gene expression (GEX, y-axis) data. **b** ALLIUM stratification into subtype and tier group for the 154 B-other patients. **c** Subtype distribution after molecular and ALLIUM re-classification for the complete set of 281 patients with unclassified subtype at the start of the study. **d** Unsupervised dimensionality reduction (UMAP) based on the DNAm levels of 379 CpG sites across the 971 samples with molecularly defined subtype and 139 controls used to train ALLIUM DNAm and the 102 B-other samples reclassified by ALLIUM DNAm. **e** UMAP based on 356 genes across 248 samples with molecularly defined subtype and 12 controls used to design ALLIUM GEX and the 56 B-other samples reclassified by ALLIUM GEX. **f** Flow chart of molecular subtype revision in the study. **g** Distribution of subtypes across entire BCP-ALL cohort (*n* = 1025) color-coded by subtype determined at ALL diagnosis (start of study) and **h** distribution after molecular screening and ALLIUM re-classification (end of study).

Next, we applied ALLIUM to the 154 remaining B-other cases in our cohort (Supplementary Data 23–24). For 67 cases where both DNAm and GEX data were available, 51 (76.1%) received concordant subtype predictions (Fig. 5a). The highest concordance was observed for the prediction of subtypes with fusion genes, i.e. *ZNF384* (4/4, 100%), *KMT2A* (1/1, 100%), *DUX4*-r (11/12, 91.7%), *ETV6*::*RUNX1-like* (6/7, 85.7%), and PAX5alt (20/24, 83.3%). To establish consensus molecular subtypes for the B-other group, we constructed a 4-tier system to improve the confidence of subtype re-annotation (Fig. 5b). Tier 1 included 28 patients with a high score from the DNAm or GEX classification combined with molecular evidence to support the subtype: expressed fusion gene, CNA, karyotype, or mutation. Tier 2 comprised 34 patients with concordant GEX and DNAm classification, but lacked conclusive molecular evidence. Tier 3 included 40 patients with only DNAm predictions or a discordant prediction with one non-class and one high-score subtype prediction. Lastly, tier 4 included 52 patients where ALLIUM generated low confidence predictions or two conflicting predictions. Figure 5c illustrates the distribution of molecular subtypes within the group of 281 initially unclassified BCP-ALL patients following molecular screening and ALLIUM classification (tier 1-3).

The reclassified samples clustered with samples of known subtype (Fig. 5d, e, Supplementary Fig. 13). Sub-clusters were observed for *KMT2A*-r*, DUX4*-r, and T-ALL. In concordance with previous reports^46,47^, these included two putative *KMT2A*-r and *DUX4*-r subclusters based on GEX data and two T-ALL clusters in the DNAm data. Notably, the *KMT2A*-r and *DUX4*-r clusters were not visible in the DNAm visualization, while the T-ALL cluster was not visible in the GEX data, but this may be due to few (*n* = 19) patients with RNA-seq data. We examined fusion gene usage and found that the *KMT2A-r* cluster 1 (C1, *n* = 5) was characterized by *USP2* (*n* = 3) and *USP8* (*n* = 1) fusions, while C2 (*n* = 7) primarily contained patients with *KMT2A*::*AFF1* fusions (*n* = 4), indicating sub-clustering associated with the fusion partner. The patients in *DUX4*-r cluster 1 (C1, *n* = 15) expressed *DUX4*::*IGH* (*n* = 10), alongside a diverse array of other fusions, including *CRLF2*::*IRF1*, *PAX5*::*FLI1*, *ELL*::*KLF2*, *ATAD2*::*NPM1* and *PAX5*::*FOXP1*. In *DUX4*-r cluster 2 (C2, *n* = 16), *DUX4*::*IGH* (*n* = 9) was the most prevalent fusion. Seven of the 19 T-ALL patients with RNA-seq data carried fusion genes, but no apparent clustering by fusion partner was observed. Additional information about the fusion genes detected by group can be found in the Supplementary Fig. 14. In summary, by employing molecular screening and the ALLIUM classification method, we successfully elucidated the molecular subtype for 229 out of 281 BCP-ALL patients who had not been previously characterized (Fig. 5f). Consequently, our study expanded the scope of known subtypes across the entire cohort, encompassing a total of 973 out of 1025 BCP-ALL patients (94.9%) (Fig. 5g, h).

### Clinical characteristics of molecular subtypes

**Fig. 6:**
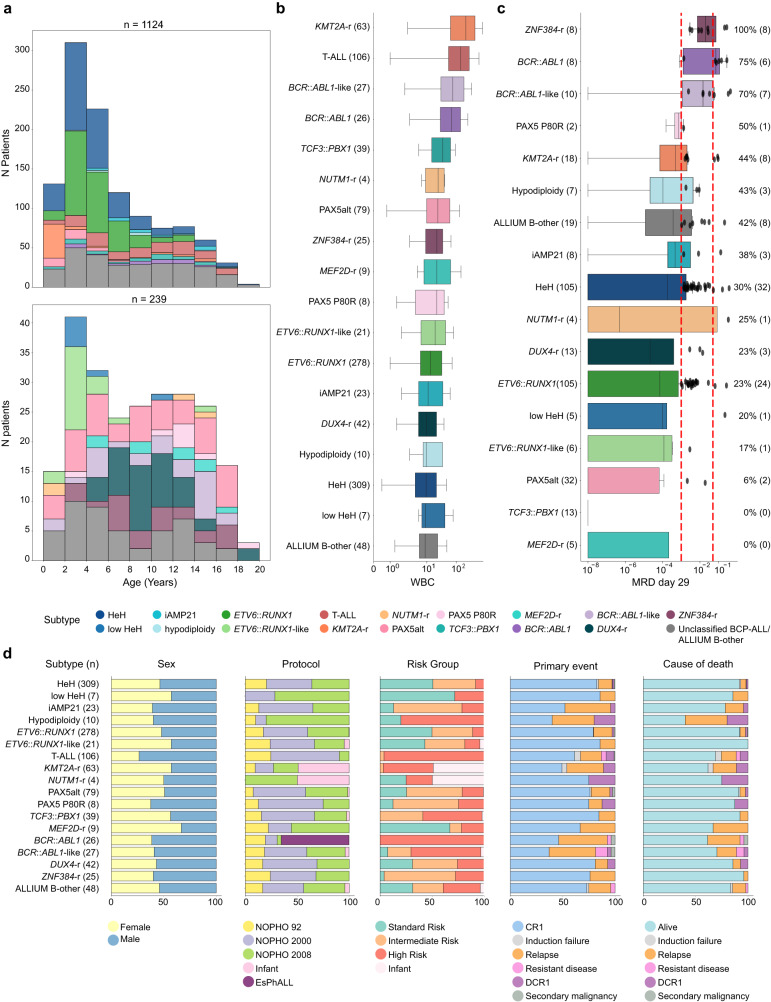
Clinical variables by molecular subtype of 1124 patients with clinical data available. **a** Histogram of subtype distribution by age. The age distribution color coded by subtype determined at ALL diagnosis is indicated in the top panel. The distribution of the originally unclassified patients color coded by revised molecular subtype is indicated in the lower panel. **b** Boxplots of the white blood cell count (WBC) at ALL diagnosis by revised molecular subtype. **c** Boxplot of minimal residual disease (MRD) levels at day 29 of treatment, for 368 patients with MRD information available. **d** Stacked barplots showing sex, treatment protocol, risk groups, primary event, and cause of death per subtype by reclassified subtype. CR1: complete remission, DCR1: death in complete remission, smn: secondary malignant neoplasm. The plots are color-coded based on their respective subtypes. The lines (whiskers) on the boxplots represent the distribution of residual data points beyond the lower and upper quartiles.

We obtained complete clinical data from 1124 out of the 1131 patients in our study cohort. The median follow-up period for surviving patients was 16.0 years (interquartile range, IQR, 13.0–19.0). As anticipated, the basic clinical variables exhibited significant variation across different subgroups and these closely aligned with well-established patterns observed in other well-described ALL cohorts (Supplementary Data 25). The distribution of ALL subtypes based on patient age at the time of diagnosis is similar to other cohorts (Fig. 6a)^1^. The white blood cell (WBC) count also demonstrated significant variability among subtypes. High-risk subtypes, including T-ALL, *KMT2A*-r, *BCR*::*ABL1*, and *BCR*::*ABL1*-like ALL, were associated with elevated WBC counts, whereas patients with established low-risk subtypes, such as HeH and *ETV6*::*RUNX1*, exhibited the lowest WBC levels. Notably, the *DUX4* group consistently displayed the lowest WBC counts across all subgroups (Fig. 6b).

Minimal residual disease (MRD) status at the end of the induction phase (day 29) exhibited notable variation among the subtypes (Kruskal-Wallis p-value = 1.36e-08, Fig. 6c). For instance, the majority of patients with *MEF2D*-r (*n* = 5/5, 100%), *TCF3*::*PBX1* (*n* = 13/13, 100%), and PAX5alt (*n* = 30/32, 93.8%) were MRD-negative at day 29. In contrast, all eight *ZNF384*-r cases were MRD-positive. Intriguingly, despite the high MRD levels observed in the *ZNF384*-r analyzed by MRD, only six relapses and one death were recorded in this subgroup. Patients belonging to the *ETV6*::*RUNX1*-like and *PAX5*-alt groups also demonstrated favorable overall outcomes, with >75% achieving complete remission without events. In line with expectations, emerging high-risk subtypes, such as *BCR*::*ABL1*-like, presented poor outcomes.

## Discussion

Recent developments in integrated large-scale genomic analyses have greatly improved our knowledge of the genetic basis of ALL, identification of new subtypes and disrupted pathways that can be targeted therapeutically^20,24,46,48^. Accurate detection of the subtype-defining alterations in the clinical setting is crucial to guide risk and treatment stratification, monitor treatment response, and is very important for future implementation of tailored or precision therapy^2^. Given the low frequencies of rare subtypes in ALL and the long follow-up data needed to evaluate their clinical relevance, it is imperative to have methods that allow for retrospective analysis of biobank material, in addition to robust diagnostics in prospective cases. Herein, we designed and implemented a multimodal classification approach for ALL (ALLIUM) that captures epigenomic and transcriptomic alterations left as a detectable footprint in ALL cells. We demonstrate the utility of ALLIUM by retrospectively evaluating the frequency and clinical impact of emerging molecular cytogenetic subtypes in a large cohort of patients treated uniformly on NOPHO protocols between 1996 and 2013 and in external datasets.

Machine learning (ML) has the potential to improve clinical diagnostics by enabling automated and accurate diagnostics, with reduced cost^49^. A unique feature of ALLIUM, over other ML-based subtype algorithms^26,27^ is that it can use multiple modalities (DNA methylation and/or gene expression) for subtype determination. We demonstrate that a DNAm-based classifier can achieve a comparable performance to GEX-based methods. A specific strength of DNAm as an analyte is its ability to identify disease-related methylation patterns and potential biomarkers in archived samples^33^. By using biobank samples and retrospective cohort studies, insight into long-term disease outcome can be gained, which would be difficult to obtain through prospective study designs, especially for rare subtypes. One limitation of the DNAm classifier was its reduced sensitivity, which can be ascribed to the inherent constraint of DNAm data, which ranges from 0% to 100% methylation per CpG site. This limited range renders DNAm data more susceptible to confounding effects stemming from lower blast percentages. In contrast, the dynamic range inherent in gene expression (GEX) may offer greater flexibility, enabling compensation or correction in scenarios involving low blast percentages^27^. The ability of RNA-seq to detect fusion genes and coding mutations that can provide clear molecular evidence for subtype decision making, gives additional value to the GEX approach^26,27^ for prospective clinical diagnostics. However, RNA is not as readily available from historical material in biobanks, limiting the usefulness of GEX classifiers for retrospective interrogations. On the other hand, immerging studies demonstrate how DNA methylation holds significant potential for prognostication across a spectrum of hematological malignancies^47,50–53^, which will be an interesting avenue to pursue in future studies. Array-based DNAm assays also have the added advantage of concurrently generating comprehensive CNA profiles^54^. These CNA profiles help distinguishing subtypes characterized by large-scale copy number changes, such as HeH, low HeH, hypodiploidy and iAMP21. In centers where DNAm subtyping for brain cancer is already established^55,56^, the incorporation of ALLIUM DNAm subtyping could serve as a complementary diagnostic modality.

Using ALLIUM as a tool, we were able to accurately detect molecular ALL subtypes for up to 81.5% of previously unclassified BCP-ALL cases in our population-based Nordic cohort spanning three NOPHO protocols (1992, 2000, 2008). We found that the molecular composition of BCP-ALL cases in the Nordics is comparable to studies from Europe^57,58^, USA^20,46,59^, and Asia^60^, and others^61^. In order of prevalence, these include PAX5alt (with a frequency of 8% compared to a range of 4–10% in the aforementioned studies), *BCR*::*ABL1*-like (3% vs 3–13%), *DUX4*-r (4% vs 4–7%), *ETV6*::*RUNX1*-like (2% vs 1–3%), *MEFD2*-r (<1% vs 1–2%), *NUTM1*-r (<1%, vs < 1–1%), PAX5 P80R (<1% vs 1–2%). ALLIUM performed comparably to two other GEX subtyping models^26,27^, with two differences. Due to lack of training samples from rare subtypes, including t(5;14)(q31.1;q32.3)/*IL3*::*IGH*, *IKZF1* N159Y, and *CDX2*::*UBTF*^62^ in the Nordic training set, ALLIUM cannot differentiate these subtypes. A second notable difference between ALLIUM and the other models is their performance distinguishing PAX5alt from *BCR*::*ABL1*-like. This may be due to differences in how these subtypes were defined in the training sets. *PAX5* alterations have been carefully curated in the Nordics, based on a combination of PCR and high-resolution genome-wide analyses^21,42,63,64^. For example, the *PAX5*-driven aberration dic(9;20) was included as an obligatory risk-stratifying subgroup in the NOPHO-2008 protocol, and thus this aberration has been studied in detail^38,65^. The low-risk clinical indicators (low WBC, MRD-, 76% EFS) in the PAX5alt group in combination with high-risk indicators (high WBC, MRD + , 37% EFS) in the *BCR*::*ABL1*-like group would indicate that the groups defined by ALLIUM distinguish potentially clinically relevant groups. Further clarification will be needed in future efforts to refine subtype-decision models and highlights the need for large international collaborations to achieve this.

An additional strength of our study is our ability to assess the added value of MRD risk stratification in light of new molecular subtypes^37^. Although MRD remains as one of the best prognostic markers for treatment outcome in ALL, our results further underscores that MRD stratification in the new subtypes may not be uniformly applicable^59,60,66^. Furthermore, early monocytic lineage switching, which includes loss of the B-cell immunophenotype, has been described in *DUX4*-r, *ZNF394*-r and PAX5 P80R subtypes^66^, potentially leading to an underestimation of MRD levels in these groups. However, questions still remain if MRD is a clinically relevant measure for treatment decisions in these new groups. Although we do not know what the MRD levels were of the patients included herein treated prior to 2008, our confirmatory observations further support that slow clearance of MRD specifically in the *ZNF384*-r may not accurately measure future outcome.

Our study primarily focused on molecular subtype classification. While our study provides valuable insights into molecular subtypes, we did not comprehensively analyze correlations with clinical data, treatment regimens, or long-term follow-up information. This lack of comprehensive clinical investigation limits our ability to draw conclusions about the impact of specific subtypes on treatment responses and long-term outcomes, which will be important to address in future studies. Finally, our study is based on the current state of knowledge and the 17 of the 22 know ICC subtypes that were present in our Nordic training cohort. The field is rapidly evolving, with new subtypes, biomarkers, and machine learning models adapted for this purpose^26,27,67,68^. The development of multiple ALL classification methods ensures that solutions are well-tested, adaptable, and capable of addressing a wide array of samples and cohorts from diverse genetic backgrounds. Although ALLIUM was not trained on all ICC subtypes, it remains the only model available for predicting ALL subtypes from DNA methylation data.

In summary, by implementing ALLIUM for retrospective analysis of a large retrospective ALL cohort, we were able to accurately assess molecular subtype in previously undefined Nordic BCP-ALL cases. ALLIUM is freely available on GitHub and can be applied to determine molecular subtype membership of patients with either DNA methylation array data or RNA-seq data for research, or to support future precision diagnostics in pediatric ALL.

## Methods

### Patients

Bone marrow aspirates or peripheral blood samples collected at diagnosis from 1131 unique population.-based pediatric ALL patients were obtained from children diagnosed in the Nordic countries during 1996–2013 and enrolled on the Nordic Society of Pediatric Hematology and Oncology (NOPHO) NOPHO-92 (*n* = 201), NOPHO-2000 (*n* = 493), NOPHO-2008 (*n* = 380), EsPh-ALL (*n* = 17), or Interfant (*n* = 40) treatment protocols^34–37^. Molecular diagnosis of ALL was established by analysis of leukemic cells at the time of diagnosis with respect to morphology, immunophenotype and cytogenetics. The guardians and/or the patients provided written consent. The study was conducted according to the guidelines of the Declaration of Helsinki and approved by the Regional Ethical Review Authority in Uppsala, Sweden and by the NOPHO Scientific Committee (Study #56).

### DNA and RNA extraction

DNA and RNA were extracted from primary ALL cells after Ficoll gradient separation using reagents from the AllPrep DNA/RNA/miRNA Universal Kit (Qiagen) or the AllPrep DNA/RNA Kit (Qiagen), including a DNase treatment step (Qiagen). DNA and RNA were quantified using the reagents from the double-stranded DNA Broad Range Kit or the RNA Broad Range kit on a Qubit instrument (Life Technologies). RNA quality was determined using the RNA Integrity Number (RIN) assessed by the Bioanalyzer or TapeStation system (Agilent).

### DNA methylation arrays

Genome-wide DNA methylation levels were determined using the Infinium HumMeth450K BeadChip assay (450k array, Illumina). DNAm data were generated using 250 ng input DNA from 384 newly collected BCP-ALL samples on the 450k array. Data from 741 patients were retrieved from Gene Expression Omnibus (GEO) entry GSE49031^45^. Batch correction was not applied to the DNAm data (Supplementary Fig. 15). The complete DNAm dataset (1125 patients) was firstly filtered according to a previous study^45^ resulting in 435,941 CpG sites and then to include probes present on the MethylationEPIC v.1.0. B5 manifest file (https://emea.support.illumina.com/downloads/infinium-methylationepic-v1-0-product-files.html), resulting in 406,542 CpG sites. After variance-based filtering (variance < 0.01), 167,353 CpGs remained for downstream analysis. CNAs were detected using intensity levels from the 450k arrays using the R package “CopyNumber450kCancer”^69^. Data from 50 normal blood cell samples (CD3 + and CD19 + , GSE49031) was used as control data for normalization and transformation of probe intensities (log2 ratio, LogR).

### RNA sequencing

A total of 328 samples from 315 patients were subjected to RNA sequencing (Supplementary Data 2). RNA sequencing libraries were prepared from 132 samples with RIN > 7 using the Illumina TruSeq stranded Total RNA (RiboZero human/mouse/rat) kit with 300 ng of total input RNA. The libraries were paired-end (PE) sequenced (150 bp) on an Illumina HiSeq2500 or NovaSeq 6000 instrument to an average of 49.8 M (range 30.2-113.2 M) PE 150 bp reads per sample. Samples with RIN < 7 or with less than 300 ng input RNA available were prepared with the Illumina TruSeq RNA Access library preparation kit (*n* = 28 samples) and sequenced on an Illumina HiSeq 2500 instrument PE 150 bp to an average 34.0 M (range 12.6-55.1 M). RNA-seq data from 162 samples, generated with 1000 ng input RNA using the Script-Seq kit (EpiCentre)^9,39,70^ and 6 samples prepared with Illumina RNA access protocol^39,41^ were collected from previous studies. The raw sequencing data for each of the 328 libraries included in the study were processed together using the nextflow-based (21.02.0.edge) nf-core/rnaseq (3.0) pipeline, which includes trimming of the paired-end reads by trimgalore (0.6.6), alignment to GRCh38.103 with STAR (2.6.1d). The aligned reads were quantified at the transcript level using Salmon (1.4.0) and the transcript level expression values were subsequently summarized to the gene level using the bioconductor package tximeta (1.8.0). The gene count matrix was corrected for batch effects with ComBat-Seq. The genes were subsequently filtered to remove Y chromosome, scaffold, mitochondrial (MT), and ribosomal (RPS and RPL) genes, as well as non-protein coding genes resulting in 19,774 protein-coding genes for downstream analysis. Data were normalized using Gene Length corrected trimmed mean of M-values (GeTMM), adjusting the data for both gene length and library size and finally log2 transformed. Technical (*n* = 5, repeated RNA-seq library construction from same RNA sample) and biological replicates (*n* = 8, sample taken at relapse) from 11 patients were used to validate merging the different library types (Supplementary Fig. 15).

Fusion genes were detected using a combination of FusionCatcher 0.99.7d^71^ and targeted screening of 22 genes known ALL fusions (Supplementary Data 2). Fusion gene status for 61 patients in the study were described previously^7^. Candidate fusion genes were validated by supporting karyotype data, copy number analysis and/or by experimental validation using Sanger sequencing as previously described^9^.

### Mutational analysis

Somatic single nucleotide variants (SNVs) were retrieved from a 872-cancer gene Haloplex panel for 144 patients in our study cohort^72,73^ and from whole genome sequencing performed on 41 patients^40,41,72^. Variant alleles *PAX5* p.Pro80Arg, *IKZF1* p.Asn159Tyr, and *ZEB2* p.His1038Arg were screened for in the 328 samples with RNA-seq data using alleleCount/3.2.2 (https://github.com/cancerit/alleleCount) on bam files.

### ALL subtype classification

ALLIUM was built using the scikit-learn package, based on the Nearest Shrunken Centroid (NSC) method^44,74^. Classifiers were built for each of 17 established molecular ALL subtypes present in our cohort and for healthy controls in a supervised manner. Models for DNAm and GEX datasets were designed separately. First, the data were split into design (known subtypes), hold-out (known subtypes) and discovery (B-other) sets. The models were trained, optimized and features were selected on the design set and then their performance was evaluated on hold-out and internal replication datasets. The models were further validated in independent external validation datasets: RNA-seq data from 65 Finnish patients from GSE228632 for which detailed information can be found in the Supplementary Materials and Methods, and published datasets from RNA-seq of 19 BCP-ALL patients from GSE161501^43^ and 450k DNAm from 227 BCP-ALL patients GSE56600^31^. Additional details can be found in the Supplementary Materials and Methods. ALLSorts and ALLCatchR were run on the corrected count matrix (n genes = 60,666) according to the instructions (https://github.com/Oshlack/ALLSorts/wiki/1.-Installation, https://github.com/ThomasBeder/ALLCatchR)^26,27^.

### Cross-decomposition and enrichment analysis

Cross decomposition was performed with the Partial Least Squares (PLS) Canonical analysis using the scikit-learn package (sklearn.cross_decomposition) in Python to quantify the covariance between the DNA methylation and GEX datasets. PLS analysis used data from 201 patients with known molecular subtype with both data modalities (DNAm and RNA) available. The analysis was performed on the unselected set of CpG sites (*n* = 167,353) and genes (19,774), as well as the CpG sites (*n* = 379) and genes (*n* = 356) selected by ALLIUM. The patients were randomly split into a train (*n* = 134) and test data set (*n* = 67). Two components for the PLS Canonical transformer were chosen. Pearson’s correlation coefficient was used to measure the correlation between the two modalities for each component. One proportion z-tests followed by Benjamini-Hochberg correction were performed to measure the enrichment of the genomic locations of CpG sites and genes selected by ALLIUM in comparison to the unselected set of CpG sites and genes.

### Clinical data

Outcome data for the 1131 patients in the study was retrieved from the NOPHO leukemia database in February 2022. In total, 1124 patients had complete follow-up data available and the average time since diagnosis was 16.5 years (range 9-26). OS was calculated as the time from the date of diagnosis to the date of last follow-up or death of any cause. Kruskal-Wallis H-test from the Python library scipy.stats assessed the significance of subtype-stratified MRD distributions. A *p* value < 0.05 (2-tailed) was considered statistically significant.

### Supplementary information

Supplementary Information

Supplementary Data

## Data Availability

The public data were available from their original studies and available at the Gene Expression Omnibus (GEO) under accession numbers GSE49031, GSE56600, GSE228632, and GSE161501. The GEX data are available under GSE227832. The 450k DNA methylation data are available under controlled access via 10.17044/scilifelab.22303531 (https://figshare.scilifelab.se/). Requests for data sharing may be submitted to Jessica Nordlund (jessica.nordlund@medsci.uu.se).

## Code availability

All scripts and environment requirements to reproduce the analyses, as well as the ALLIUM model are available at GitHub https://github.com/Molmed/Krali_2023.
