# Supplementary material for: Multimodal classification of molecular subtypes in pediatric acute lymphoblastic leukemia
Source: NPJ Precis Oncol. 2023 Dec 8;7:131. doi: 10.1038/s41698-023-00479-5 (PMC10709574; doi:10.1038/s41698-023-00479-5)
Supplement: Supplementary file 1 — Supplementary Information [file 41698_2023_479_MOESM1_ESM.pdf]

Multimodal classification of molecular subtypes in pediatric acute lymphoblastic leukemia

Olga Krali[1], Yanara Marincevic-Zuniga[1], Gustav Arvidsson[1], Anna Pia Enblad[1,2], Anders Lundmark[1], Shumaila Sayyab[1], Vasilios Zachariadis[3], Merja Heinäniemi[4], Janne Suhonen[4], Laura Oksa[5,6], Kaisa Vepsäläinen[7], Ingegerd Öfverholm[3,8,9], Gisela Barbany[8,9], Ann Nordgren[8,9], Henrik Lilljebjörn[10], Thoas Fioretos[10], Hans O. Madsen[11], Hanne Vibeke Marquart[11,12], Trond Flaegstad[13,20], Erik Forestier[14,20], Ólafur G Jónsson[15,20], Jukka Kanerva[16,20], Olli Lohi[5,6,20], Ulrika Norén-Nyström[17,20], Kjeld Schmiegelow[18,20], Arja Harila[2,20], Mats Heyman[19,20], Gudmar Lönnerholm[2,20], Ann-Christine Syvänen[1] and Jessica Nordlund[1]*

1. Department of Medical Sciences, Molecular Precision Medicine and Science for Life Laboratory, Uppsala University, Uppsala, Sweden
2. Department of Women's and Children's Health, Uppsala University, Uppsala, Sweden
3. Department of Oncology-Pathology, Karolinska Institutet, Stockholm, Sweden
4. Institute of Biomedicine, School of Medicine, University of Eastern Finland, Kuopio, Finland
5. Tampere Center for Child, Adolescent and Maternal Health Research, Faculty of Medicine and Health Technology, Tampere University, Tampere, Finland
6. Tampere University Hospital, Tays Cancer Center, Tampere, Finland
7. Department of Pediatrics, Kuopio University Hospital, Finland
8. Department of Molecular Medicine and Surgery and Center for Molecular Medicine, Karolinska Institutet, Stockholm, Sweden
9. Department of Clinical Genetics, Karolinska University Hospital, Stockholm, Sweden
10. Division of Clinical Genetics, Dept. of Laboratory Medicine, Lund University, Lund, Sweden
11. Department of Clinical Immunology, Copenhagen University Hospital Rigshospitalet, Denmark
12. Department of Clinical Medicine, Faculty of Health and Medical Sciences, University of Copenhagen, Copenhagen, Denmark
13. Department of Pediatrics, Tromsø University and University Hospital, Tromsø, Norway
14. Department of Medical Biosciences, University of Umeå, Umeå, Sweden
15. Pediatric Hematology-Oncology, Children's Hospital, Barnaspitali Hringsins, Landspitali University Hospital, Reykjavik, Iceland
16. New Children's Hospital, Helsinki University Central Hospital and University of Helsinki, Helsinki, Finland
17. Department of Clinical Sciences, Pediatrics, Umeå University, Sweden
18. Pediatrics and Adolescent Medicine, Rigshospitalet, and the Medical Faculty, Institute of Clinical Medicine, University of Copenhagen, Copenhagen, Denmark
19. Childhood Cancer Research Unit, Karolinska Institutet, Astrid Lindgren Children's Hospital, Karolinska University Hospital, Stockholm, Sweden
20. For the Nordic Society of Pediatric Hematology and Oncology (NOPHO)

*Corresponding author:

Dr. Jessica Nordlund

Box 1432, BMC

75144 Uppsala, Sweden

Telephone: +46 704250806

Table of Contents

SUPPLEMENTARY MATERIALS AND METHODS 2
CONTROL SAMPLES 2
EXTERNAL VALIDATION 3
GSE56600 3
GSE161501 3
GSE228632 3
NEAREST SHRUNKEN CENTROID CLASSIFIERS 4
Classifier Architecture during optimization 4
Classifier Architecture during training 7
Classification Predictions 7
PERFORMANCE METRICS 8
SUPPLEMENTARY RESULTS 9
INTERNAL VALIDATION FOR ALLIUM GEX 9
EXTERNAL VALIDATION FOR ALLIUM DNAM: GSE56600 9
EXTERNAL VALIDATION FOR ALLIUM GEX: GSE161501 10
EXTERNAL VALIDATION FOR ALLIUM GEX: GSE228632 10
SUPPLEMENTARY FIGURES 11
SUPPLEMENTARY REFERENCES 26

Supplementary Materials and Methods

Control Samples

The following non-leukemic samples were used as controls to differentiate ALL samples with potential low blast count from those with unknown subtype. 450k array DNA methylation (DNAm) data generated from CD19+ B-cells, CD3+ T-cells from healthy blood donors, and bone marrow/peripheral blood from ALL patients in remission were downloaded from GSE49031. RNA Sequencing (RNA-seq) data from CD19+ B-cells, CD3+ T-cells from healthy blood donors, and two ALL patient samples in remission were obtained from a previous study[1]. The control samples were processed exactly as described in the **Material and Methods** for the ALL samples.

External Validation

The following external data sets were used to validate ALLIUM DNAm and GEX: 1) 450k array data derived from 227 pediatric ALL patients from GSE56600[2]. 2) RNA-seq GEX data from 19 pediatric ALL patients from GSE161501[3]. 3) RNA-seq GEX data from 65 pediatric ALL patients from GSE228632.

GSE56600

The beta-value matrix corresponding to 227 BCP-ALL patients was downloaded from GEO. No further preprocessing was performed on this dataset.

GSE161501

The gene count matrix downloaded from GSE161501 was lifted over from the hg19 reference genome to GRCh38.103 gene annotation file based on ENSEMBL IDs. All missing genes were replaced with zeros. The data were filtered and normalized using the workflow described in the **Materials and Methods**.

GSE228632

RNA was extracted from primary ALL bone marrow or blood cells after Ficoll/Lymphoprep™ (GE Healthcare, IL, USA / Stemcell Technologies, Vancouver, Canada) gradient separation or from PAXGene Blood RNA Tubes, using PAXgene Blood RNA kit (Qiagen), GeneJet RNA Purification kit (Thermo Fisher), or AllPrep DNA/RNA/miRNA Universal Kit (Qiagen). The RNA was quantified on a Nanodrop and a Qubit instrument (Thermo Fisher). RNA sequencing was performed in four batches with library preparation protocols outlined in **Table 1**. All RNA-seq libraries were sequenced on an Illumina NovaSeq 6000 instrument. The raw sequencing data were processed using the workflow described in the **Materials and Methods**.

Table 1. Batch information in response to RNA sequencing for the NOPHO GSE228632cohort (n = 65).

Batch	RNA source/ extraction kit	Ribosomal depletion	Library prep	# Samples

**C101HW18062568**	PAXgene Blood RNA kit (Qiagen)	Globin-Zero Gold rRNA Removal Kit	NEBNext Ultra Directional RNA Library Prep Kit	24
**X204SC22010852**	ALLPrep DNA/RNA/miRNA Universal kit (Qiagen)	Ribo-Zero rRNA Removal Kit	NEBNext Ultra Directional RNA Library Prep Kit	19
**X204SC21062884**	GeneJet RNA Purification kit (Thermo Fisher) or ALLPrep DNA/RNA/miRNA Universal kit (Qiagen)	Ribo-Zero rRNA Removal Kit	NEBNext Ultra Directional RNA Library Prep Kit	13
**X201SC19081411-Z01-F002**	PAXgene Blood RNA kit (Qiagen)	Ribo-Zero rRNA Removal Kit	NEBNext Ultra Directional RNA Library Prep Kit	9

Nearest Shrunken Centroid Classifiers

The Nearest Shrunken Centroid (NSC) [4] classification approach was selected due to its ability to perform training and feature selection (FS) based on a hyperparameter called *shrink threshold*. The shrink threshold moves each class' centroid (mean) for every feature (gene or CpG site) towards zero. Features with zero or constant value across all classes (non-informative features) are not used during the prediction procedure. The goal of this workflow is to remove noise from the data and capture meaningful signatures for each subtype.

Classifier Architecture during optimization

The model was trained on the following 12 ALL groups with one or more subtype members and a control classifier comprising of healthy samples: aneuploidy (HeH, low HeH, hypodiploidy and iAMP21), *ETV6* gene rearrangements (*ETV6*::*RUNX1* and *ETV6*::*RUNX1*-like), the Philadelphia (ph) chromosome

(*BCR*::*ABL1* and *BCR*::*ABL1*-like), *TCF3*::*PBX1*, PAX5alt, PAX5 P80R, *KMT2A*-r, *ZNF384*-r, *DUX4*-r, *MEF2D*-r, *NUTM1*-r and T-ALL.

Subtype groups and one-vs-rest approach

For each subtype/group classifier, a one-vs-rest approach was implemented, where all other subtypes than the one of interest formed the 'rest' group (**Supplementary Figure 2**). This architecture allowed to select group specific signatures which separate each subtype group from all the rest. At the end of each group loop, a set of group-specific signatures (CpG sites/genes) were obtained. The rationale behind dividing a multiclass problem into multiple binary (one-vs-rest) classification tasks on the subtype group level was to identify patients who do not belong to any class or belong to multiple ones. This is in contrast to a multiclass model, which forces all patients into a single subtype, even if they do not adequately match the subtypes used to design the classifier.

Group Members

If a group contained two members (two subtypes), a one vs one approach was applied to obtain member signatures that separate one from the other, i.e. for differentiating between *ETV6*::*RUNX1* vs *ETV6*::*RUNX1*-like within the *ETV6*-group and *BCR*::*ABL1* vs *BCR*::*ABL1*-like for the ph-group. Otherwise, for groups with >2 members (i.e. The aneuploidy group) a one vs rest approach was implemented instead, leading to signatures that separate the member subtype from all the rest: HeH vs rest aneuploidy, low HeH vs rest aneuploidy, hypodiploidy vs rest aneuploidy, and iAMP21 vs rest aneuploidy (**Supplementary Figure 2**).

Classifier Optimization and Signature Selection

The training loop consisted of two cross validation (CV) loops. The outer loop performed stratified 5-fold CV repeated 5 times, resulting in 25 different train and test sets. Stratified 5-fold CV repeated 5 times took place in the inner loop as well, where each of the 25 training datasets were divided into 25 inner train and validation sets (**Supplementary Figure 2**). Stratified folds were preferred since they retained each dataset's characteristics in terms of patient representation in each subtype group after splitting the datasets into k-folds.

The DNAm dataset underwent pre-processing during model optimization. More specifically, CpG sites with >10% missing values across all patient samples were discarded, as well as sites with average b-value class-wise pair difference < 20%. Furthermore, the data were imputed for missing values using the median. These pre-processing steps took place iteratively on the outer loops by using the 25 outer train sets (**Supplementary Figure 2**). Then, the same filtering and imputation was applied on the test tests during CV.

This iterative process was repeated for each of the shrink threshold parameter values. The shrink threshold parameters varied between subtypes as the number of patients within each group varied as well (**Supplementary Data 26-27**), and applied only during model optimization to obtain the best set of features per threshold value. In the inner loop, the model was trained using the inner train sets with the selected shrink threshold and all informative features per fold were obtained. At the end of all 25 inner folds, all features regardless the number of occurrence across the inner folds were retained. Permutation importance was performed on the outer folds to ensure a more robust subtype-specific signature selection. Approaches as permutation importance are prone to fail due to collinearity. To mitigate this, Least Absolute Shrinkage and Selection Operator (LASSO) regression was implemented to remove collinear features. For the DNAm model, the alpha was set to 0.001, while for the GEX an alpha of 0.01. Features with importance scores > 0 were retained in each outer fold. The model was then trained on the outer train sets with the corresponding top features per outer fold, tested on the test sets and the test F1 score per outer fold was obtained. Finally, the consensus features were selected based on the highest test F1 score across all 25 outer folds (**Supplementary Figure 2**).

Additionally, external CV took place following the same architecture as previously described for optimization using the consensus signatures selected for each shrink threshold to obtain train, test and CV F1 scores. The data was first fitted on the inner train sets to obtain the mean CV F1 scores across all inner folds per outer fold, and then on the outer train sets to obtain both train and test F1 scores per outer fold. Finally, the mean train, CV and test F1 scores across all 25 outer folds per shrink threshold were kept

(**Supplementary Figure 2**). Then, in a final feature selection step the signatures of the threshold with the best performing model, in terms of classification overall F1 scores, were selected for each classifier. Since the difference in F1 scores was very small across shrink thresholds, a manual rule was set, where if applicable, to choose the shrink threshold that included the highest number of features but not more than 45 genes or 40 CpGs per subtype group. At the end, all signatures appearing in more than one subtypes were dropped to retain only the unique subtype-specific ones.

Classifier Architecture during training

Groups and members

Each classifier was trained either using its own signatures (DNAm) or all signatures (GEX) selected by the final feature selection step, as the DNAm and GEX classifiers performed better with subtype specific or all signatures approaches respectively. The same group-member strategy as the one mentioned during optimization was used. A one-vs- rest approach was implemented for the groups as previously described and a one-vs-one to separate one member from the other (e.g. *ETV6*::*RUNX1* vs *ETV6*::*RUNX1*-like), if the group contained two subtype members. In contrast, an overall multiclass classifier was selected for the aneuploidy members, as the subtypes were mutually exclusive there was no need for a one vs rest approach to be implemented (HeH, low HeH, iAMP21 and hypodiploid (only on the DNAm)) (**Supplementary Figure 3**).

Classification Predictions

The first part of the prediction loop consisted of the group prediction. Each sample was tested on all 12 group classifiers and the healthy classifier, and when the results highlighted aneuploidy, ETV6 or ph-group, the process continued further. Since a high group score was obtained, it was upon to the model to decide whether a sample belonged to one of the member subtypes (**Supplementary Figure 3**). The generated results provided information both in group and subtype level. The final subtype decisions were made based on the group probability scores. Each sample could receive one of the following labels: no class if ‘other’ was predicted on all group classifiers, multi-class if > 1 classes were assigned, or single class. To handle the multi-class cases, the group with the highest probability score was picked as the final group prediction,

and the subtype member with the highest score was obtained to give the final subtype prediction (**Supplementary Figure 3**). A manual check flag was raised if the probability score of the final prediction was <70%.

Performance Metrics

The classification performance metrics used included sensitivity (recall, True Positive Rate, TPR (1)), specificity (True Negative Rate, TNR (2)) and precision (Positive Predictive Value, PPV (3)). Balanced accuracy (4) was used instead of standard accuracy, since the data was imbalanced. High accuracy scores might be misleading as the performance of the under-represented class could be poor. Balanced accuracy mitigates this effect by giving an overall score taking imbalance into consideration. F1- score (5) represents the harmonic mean between specificity and precision. The closer to 1 all scores are, the better the model performs.

$$Sensitivity = \frac{TP}{TP + FN} \quad (1)$$

$$Specificity = \frac{TN}{TN + FP} \quad (2)$$

$$Precision = \frac{TP}{TP + FP} \quad (3)$$

$$Balanced\ accuracy = \frac{Sensitivity + Specificity}{2} \quad (4)$$

$$F1\ score = 2 \times \frac{Sensitivity\ \times Precision}{Sensitivity + Precision} \quad (5)$$

Finally, during model optimization to obtain the optimal signature set based on performance, F1 weighted score (6) was utilized since both comparing groups were equally important. Our classifiers did not use control (negative) vs disease state (positive) samples, thus it was of high importance to maintain good performance on both comparing groups, either it was one subtype vs all the rest, or one subtype vs another.

F1 weighted score takes into consideration all groups and calculates each F1 score multiplied by a weight (w) based on group sample size to avoid issues due to class imbalance and then averages it.

$$F1\,weighted = \frac{1}{N}\sum_{i=1}^{N} F1_i \times w_i \quad (6)$$

Supplementary Results

Internal validation for ALLIUM GEX

We internally created a validation set to evaluate ALLIUM GEX, which consisted of 13 samples from 11 unique patients (12 samples with known subtype, 1 B-other). The samples were either taken at ALL relapse (n = 8) or a new library was prepared and re-sequenced (n = 5). Ten of the 12 (83.3%) predictions matched with the true ALL subtype (**Supplementary Figure 4, Supplementary Data 9**). Eleven of the 13 predictions, in total, were concordant with the predictions of the samples taken at ALL diagnosis (**Supplementary Data 10**). The samples with mismatches include ALL_1026 (PAX5alt) and ALL_257 (*ZNF384*-r), which were taken at relapse and predicted as "no class" by ALLIUM GEX. Of interest, patient ALL_257 (*ZNF384*-r) was predicted as ETV6 group (70%) with ~50% probability to be *ETV6*::*RUNX1* (or *ETV6*::*RUNX1*-like) for the diagnostic sample (**Supplementary Data 7**). ALL_257 is known to carry characteristics of both subtypes *ZNF384*-r and *ETV6*::*RUNX1*-like [1,5].

External validation for ALLIUM DNAm: GSE56600

GSE56600 contains 450k array DNA methylation data for 227 BCP-ALL patients. ALLIUM DNAm correctly predicted 84.2% (112/133) of the patients with known subtype from this cohort. From the remaining 21 misclassified samples, 11 were classified as "control", suggesting low blast counts, and in four "no class" was predicted (**Supplementary Figure 4, Supplementary Data 11**). This cohort contained 94 patients with B-other/unknown subtype, of these ALLIUM DNAm classified 71 (75.5%) to a unique subtype, 13 with no class prediction (13.8%), and 10 as "control"/low blast count (10.6%) (**Supplementary**

Data 11). The classifier showed an overall sensitivity and specificity of 87.2% and 99.2% respectively (**Table 2, Supplementary Data 14**).

External validation for ALLIUM GEX: GSE161501

GSE161501 contained RNA-seq data for 19 BCP-ALL patients. ALLIUM GEX classifier correctly classified the subtype of each of the 19 patients in this dataset (**Supplementary Figure 4, Supplementary Data 12**), with an overall sensitivity and specificity of 100% and 100% respectively (**Table 2, Supplementary Data 14**).

External validation for ALLIUM GEX: GSE228632

RNA-seq data were available for 65 BCP-ALL samples. In total, 55 were of known subtype and 96.4% (n = 53) were correctly classified (**Supplementary Figure 4, Supplementary Data 13**). The nine B-other samples were all assigned to a subtype: *DUX4*-r (n = 3), *ETV6*::*RUNX1*-like (n =2), *ZNF384*-r (n = 2), *MEF2D*-r (n = 1) and iAMP21 (n =1). For patient ALLT-351, with hypodiploid subtype, ALLIUM GEX returned “no class” as ALLIUM GEX was not trained on this subtype. The model performance for the samples of known subtype demonstrated an overall sensitivity and specificity of 97.4% and 99.9% respectively (**Table 2, Supplementary Data 14**).

Supplementary Figures

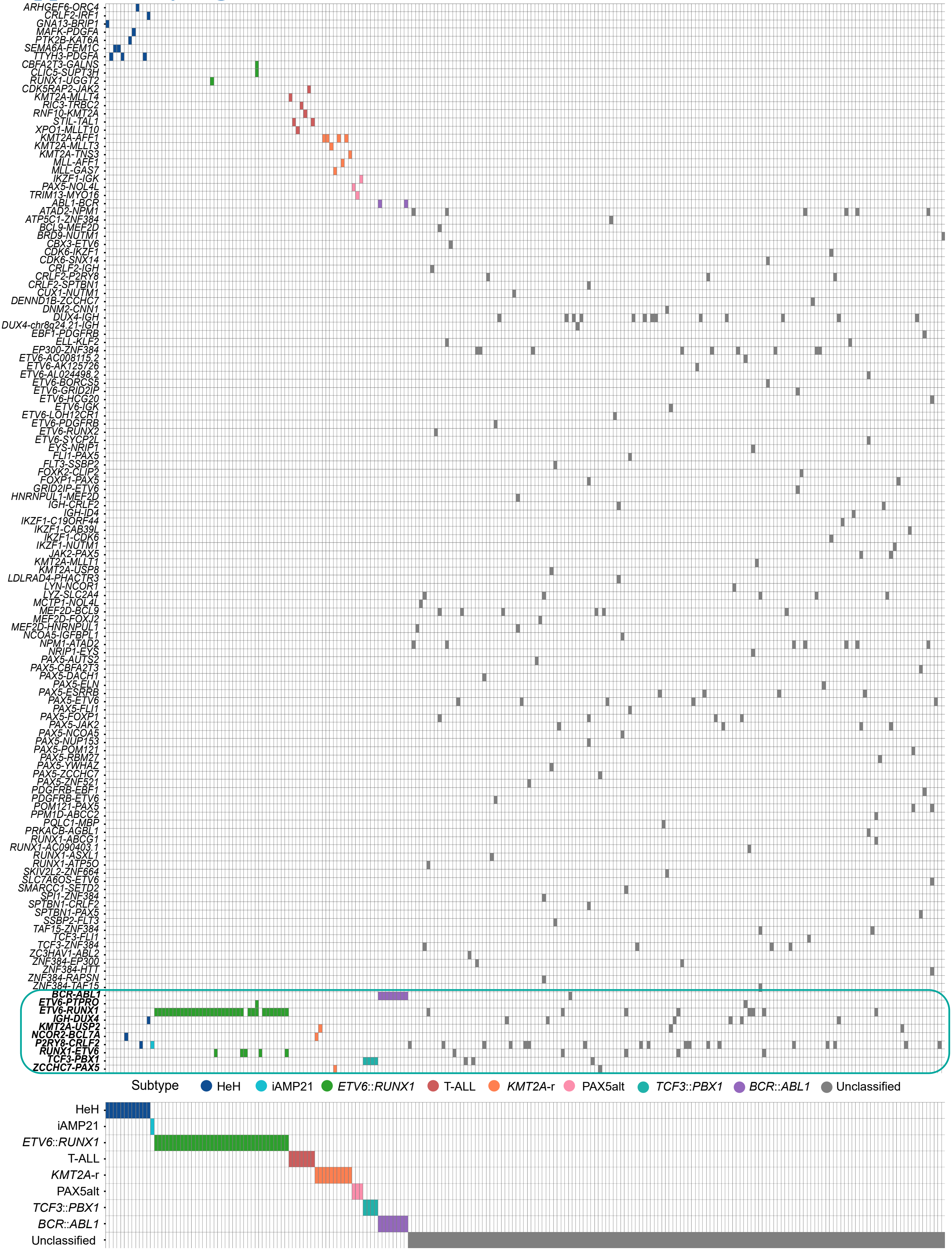

Supplementary Figure 1. Fusion gene distribution across 225 ALL patients labeled by subtype received at ALL diagnosis. The patients are denoted in columns and fusion genes in rows. In total 131 unique fusion genes (including reciprocal fusions) were detected. Ten fusion genes appeared across multiple subtypes; they are indicated within the blue rounded rectangle and denoted with bold Italics on the bottom.

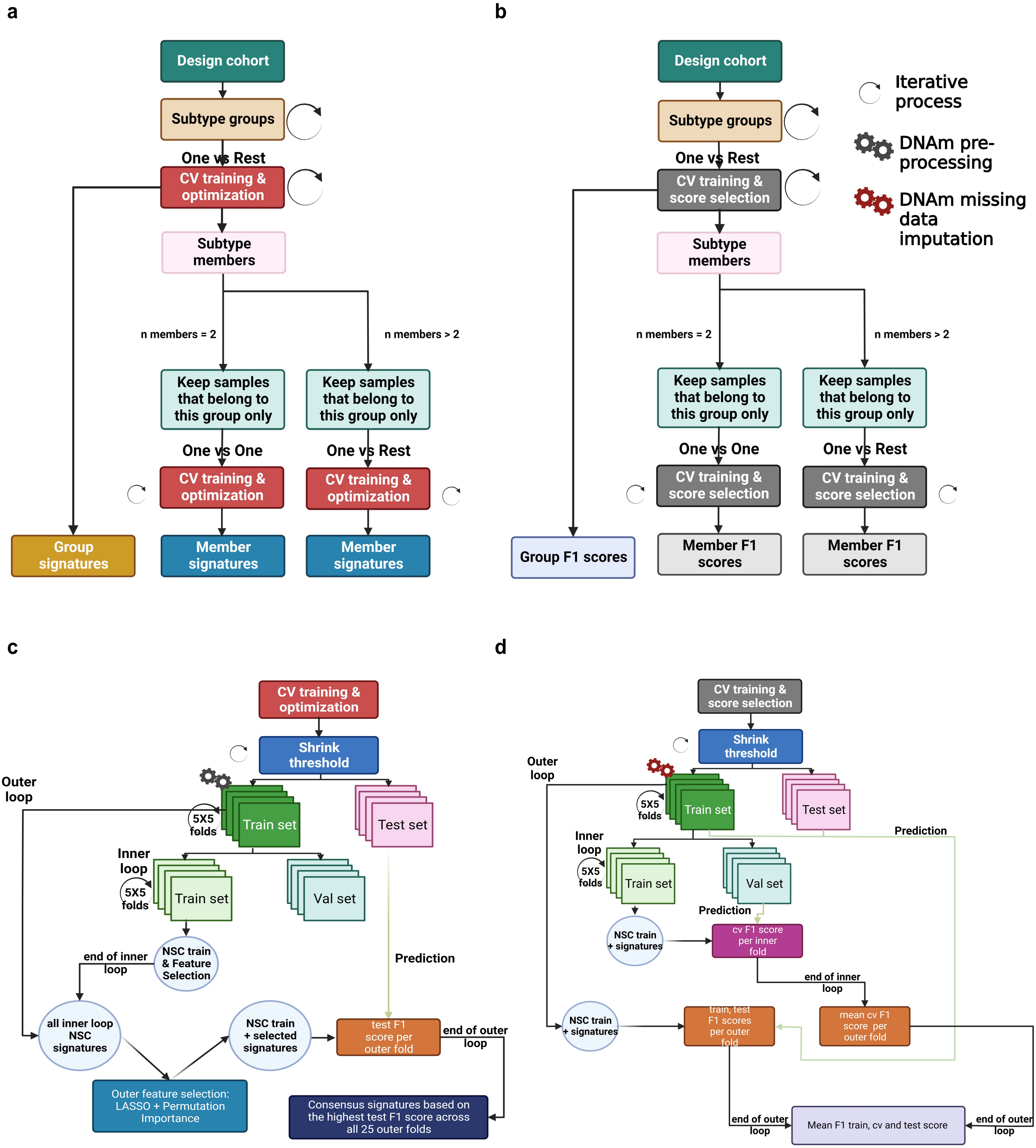

Supplementary Figure 2. ALLIUM classifier optimization and feature selection. a) Overview of cross validation (CV) and optimization iterative steps. b) Overview of CV and F1 score selection iterative steps. CV utilizes a multi-step approach for groups of subtypes with similar molecular phenotypes to obtain group signatures and subtype-specific signatures. c) During optimization, an iterative CV approach captures signatures for each shrink threshold parameter per group/subtype. d) External CV is used to obtain the train, test and CV F1 scores per shrink threshold based on the selected signatures from (c). The figure was created with BioRender.com.

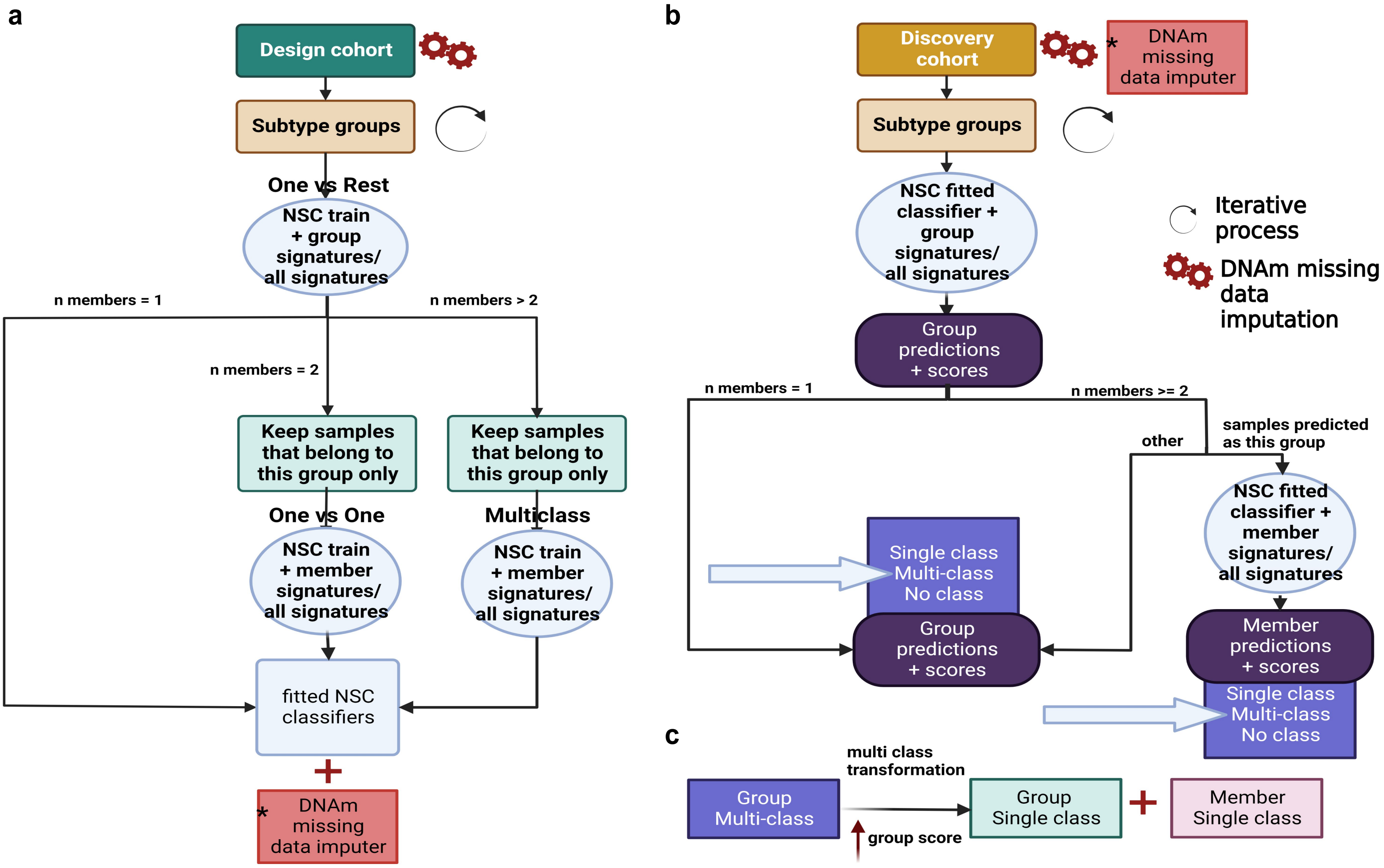

Supplementary Figure 3. Final ALLIUM classifier model architecture. a) ALLIUM was trained on all design cohort samples in a multi-step procedure including a group and a subtype membership step. When the train mode is set to 'all', all selected signatures are used to train each classifier, otherwise each classifier uses its own signatures. b) Subtype prediction is based on the trained models outlined in panel (a). c) The multi-class to single class transformation assigns subtype based on the highest group probability score. The figure was created with BioRender.com**.**

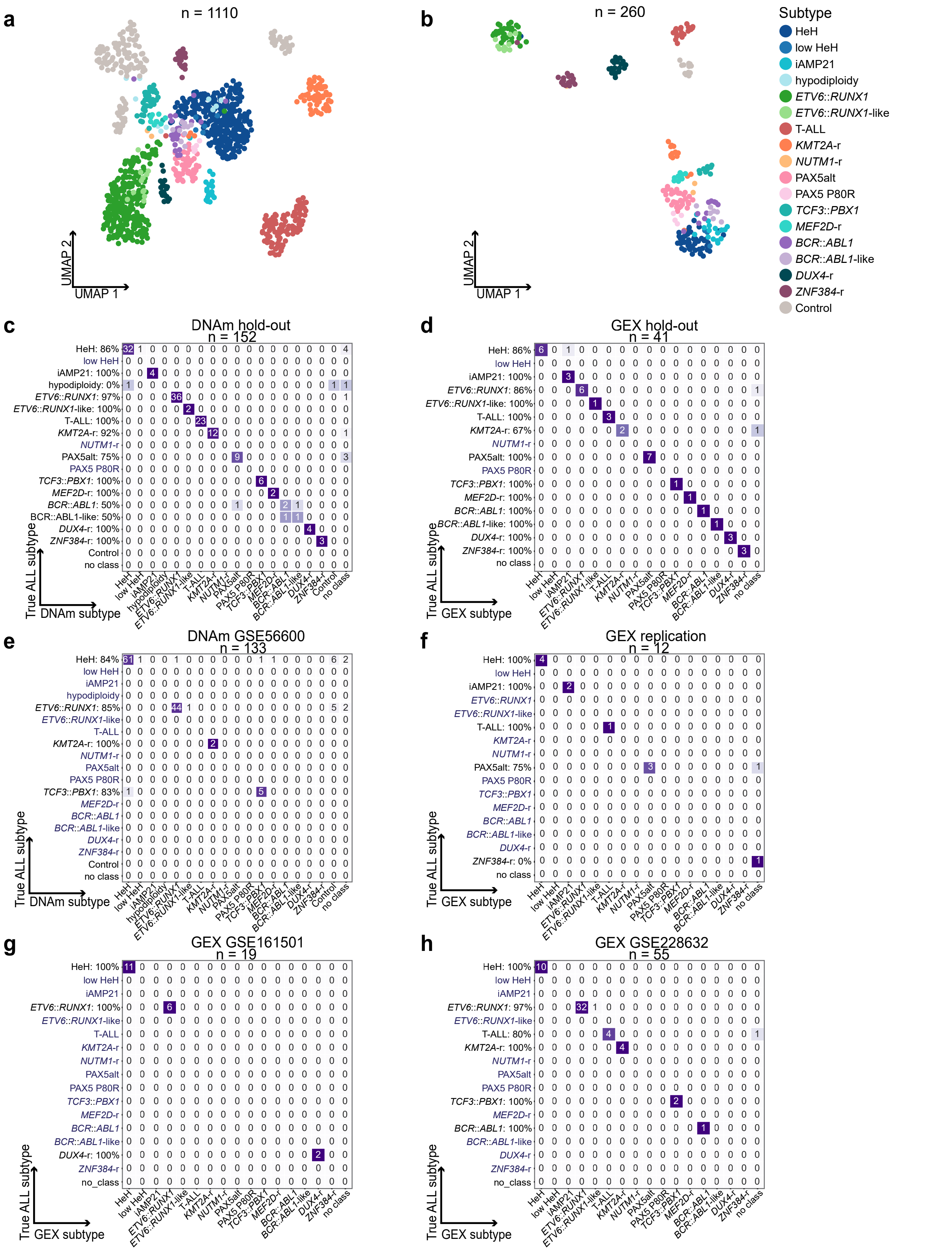

Supplementary Figure 4. ALLIUM performance evaluation. a) Unsupervised dimensionality reduction (UMAP) based on DNA methylation (DNAm) levels of 379 CpG sites across molecularly defined patients (n = 971) and controls (n = 139). b) UMAP based on gene expression (GEX) levels of 356 genes across molecularly defined patients (n = 248) and controls (n = 12). c) DNAm vs true cytogenetic subtypes for the hold-out dataset (89.5% concordance, 136/152). d) GEX predictions vs true cytogenetic subtypes for the hold-out dataset (92.7% concordance, 38/41). e) DNAm predictions vs true cytogenetic subtypes for the DNAm GSE56600 dataset (84.2 % concordance, 112/133). f) GEX predictions vs true cytogenetic subtypes for the internal replication dataset (83.3% concordance, 10/12). g) GEX predictions vs true cytogenetic subtypes for the GEX GSE161501 dataset (100%, 19/19). h) GEX predictions vs true cytogenetic subtypes for the GEX GSE228632 dataset (96.4%, 53/55). Subtypes with no patients represented are indicated by blue color on the y-axis.

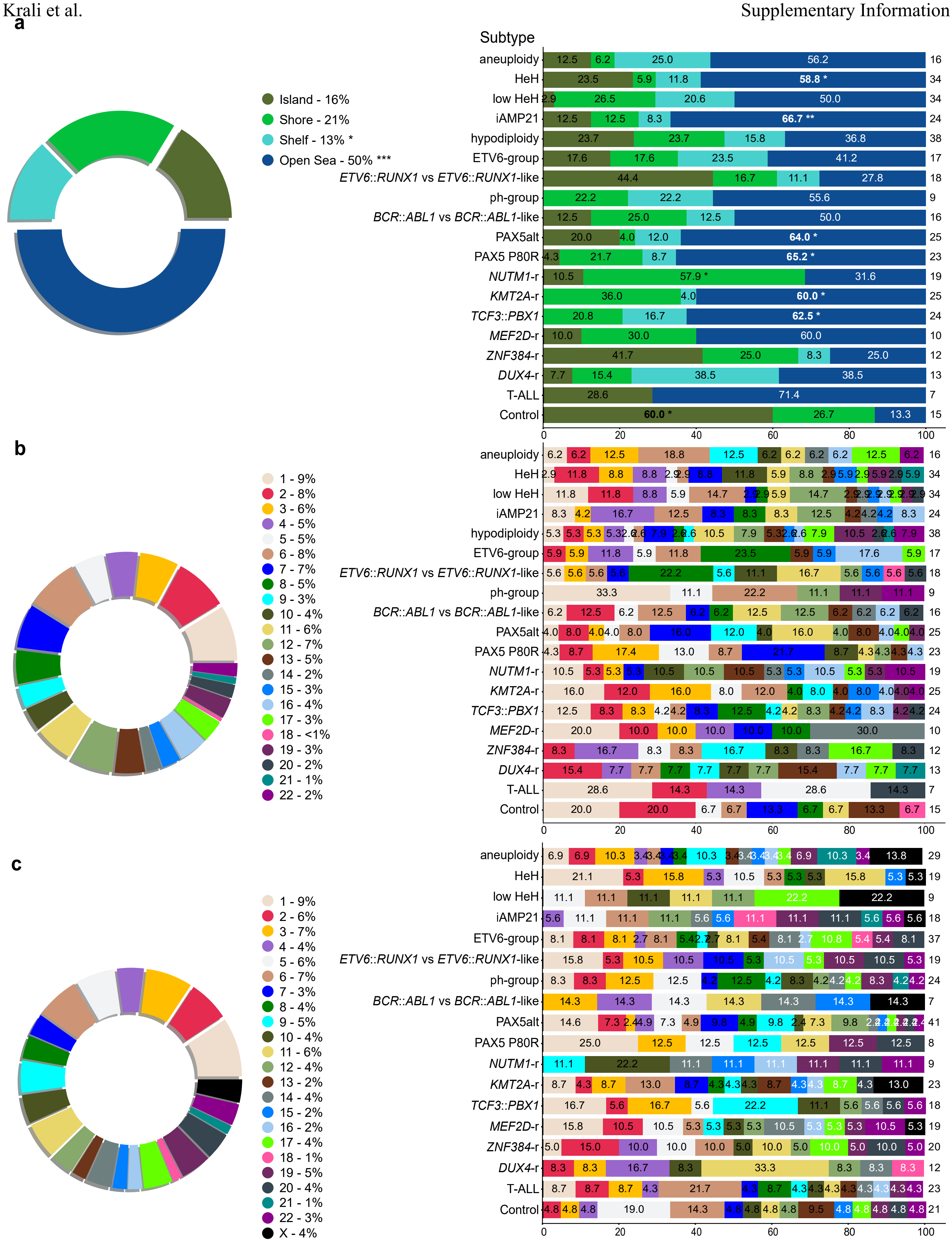

Supplementary Figure 5. Annotation of ALLIUM DNAm and GEX features. a) Vicinity to CpG islands of the 379 ALLIUM DNAm CpG sites in percentage across all subtypes (left) and per signature group (right). b) The chromosomal location of the 379 CpG sites in percentage across all subtypes (left) and per signature group (right). c) The chromosomal location of the 356 ALLIUM GEX genes in percentage across all subtypes (left) and per signature group (right). The number of signatures per group is denoted on the right of each stacked bar plot. Enrichment by the ALLIUM features are denoted with asterisk(s) (FDR p-value: *** < 0.001, ** < 0.01, * <0.05).

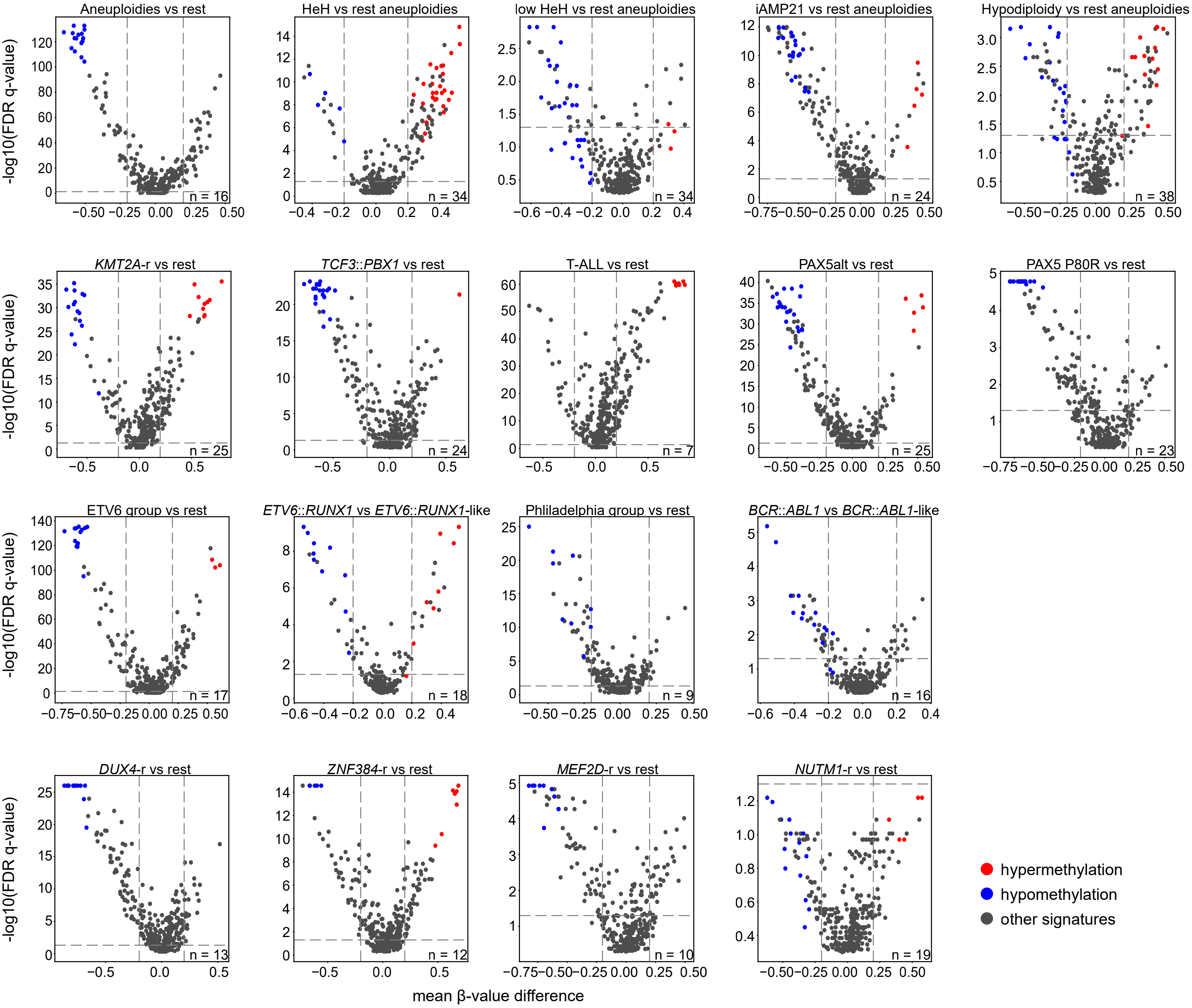

Supplementary Figure 6. Volcano plots for the CpG sites selected by ALLIUM. Mean methylation β-value difference (x-axis) is plotted against the Benjamini-Hochberg (BH) corrected Mann-Whitney U test p-value (y-axis) for the 1073 patients with established molecular subtypes after ALLIUM analysis across the 364 subtype-defining CpG sites (excl. control CpGs, n = 15). Each panel is labeled by the groups or subtypes compared and the total number of CpGs selected by ALLIUM are shown at the right bottom of the volcano plot. The CpG sites are colored according to the key to the right of the figure, where sites with β-value difference > 0 are colored in red (hypermethylated) and those < 0 are colored in blue (hypomethylated). The remaining CpG sites that were not selected to differentiate the subtype plotted are colored in grey. Signatures meeting the adjusted p-value threshold of < 0.05 and absolute β-value difference > 0.2 are located on the upper right (significantly hypermethylated) or upper left (significantly hypomethylated) rectangles defined by the threshold dashed lines.

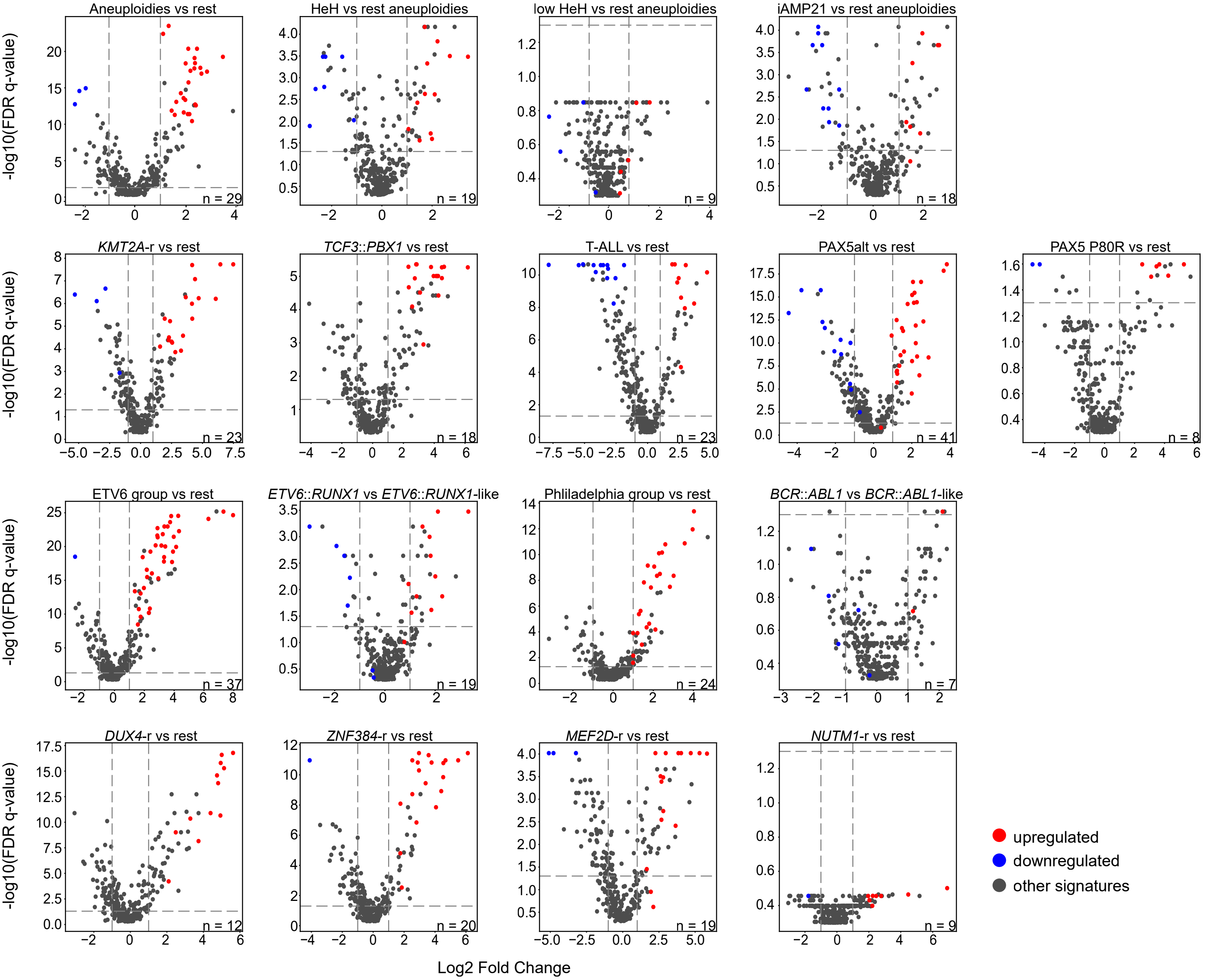

Supplementary Figure 7. Volcano plots for expressed genes determined by ALLIUM. Mean log2 fold change (log2FC, x-axis) is plotted against the Benjamini-Hochberg (BH) corrected Mann-Whitney U test p-value (y-axis) for the 304 patients with established molecular subtypes after ALLIUM analysis across the 335 subtype-defining genes high-lighted by the GEX classifier (excl. control CpGs, n = 21). Each panel is labeled by the groups or subtypes compared. The genes colored according to the key to the right of the figure, where genes with log2 change > 0 are colored in red (upregulated) and log2 change <0 in blue (downregulated). The remaining genes that were not selected for the subtype of interest are colored in grey. Signatures meeting the adjusted p-value threshold of < 0.05 and absolute log2 foldchange > 1 are located on the upper right (significantly upregulated) or upper left (significantly downregulated) rectangles defined by the threshold dashed lines.

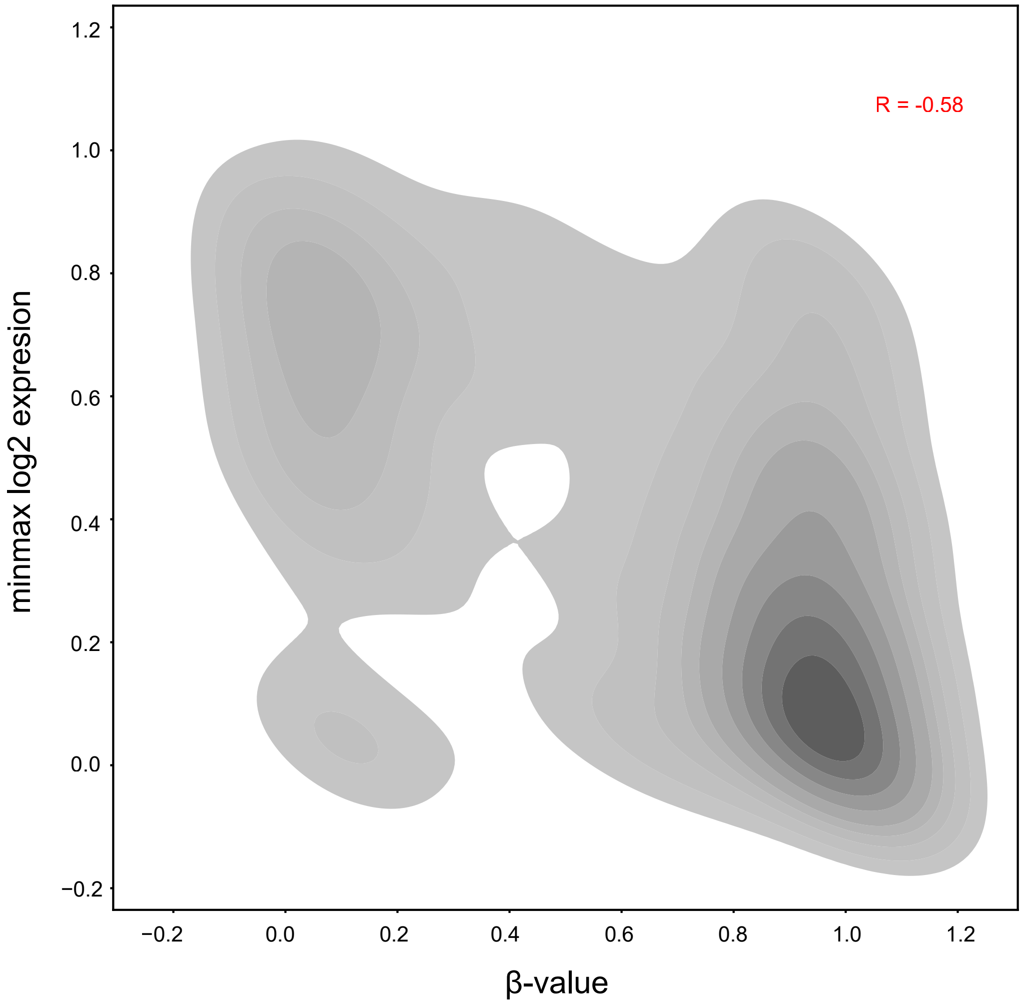

Supplementary Figure 8. Correlation between DNAm and GEX. The bivariate kernel density estimate (KDE) plot depicts the correlation of DNAm vs GEX by visualizing their distribution (β-value (x-axis) and GEX minmax scaled log2 expression (y-axis) per patient gene-CpG pair). The beta-values for eight CpGs were compared to the GEX levels of their closest mapping gene (seven genes in total) selected by ALLIUM DNAm and GEX classifiers for 201 patients with DNAm and GEX data available from the design cohort. The Pearson's correlation coefficient is indicated in the upper right corner of the figure.

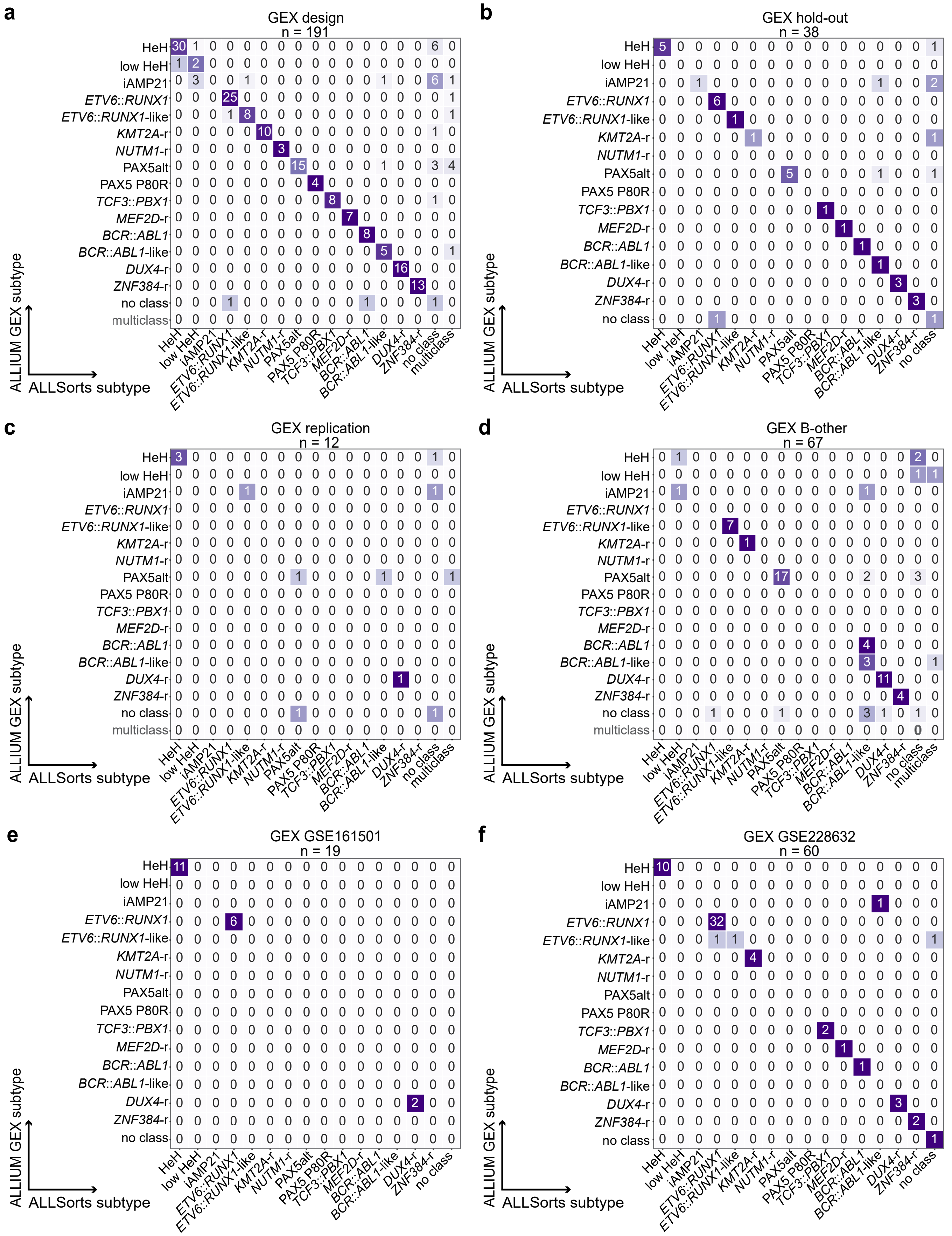

Supplementary Figure 9. Subtype predictions by ALLIUM GEX and ALLSorts[6] for GEX data. a) Design dataset 81.2% concordance (155/191). b) Hold-out dataset 78.9% concordance (30/38). c) Replication dataset 50% concordance (6/12). d) Discovery (B-other) dataset 65.7% concordance (44/67). e) GEX GSE161501 100% concordance (19/19). f) GEX NOPHO GSE228632 dataset 95.0% concordance (57/60). T-ALL patients (n = 25) were excluded. Multiclass predictions by ALLSorts are denoted in grey.

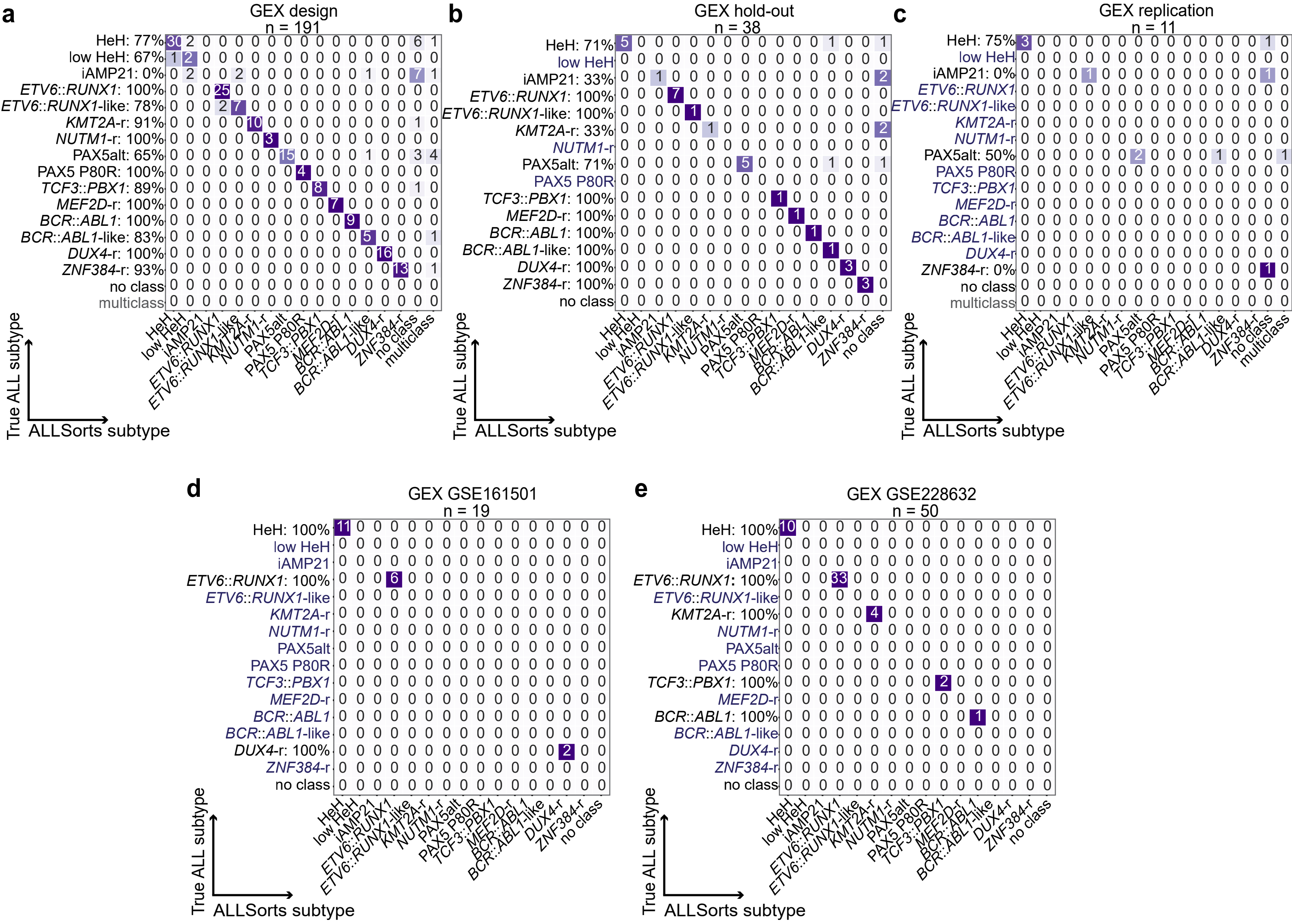

Supplementary Figure 10. Subtype predictions by ALLSorts[6] vs true cytogenetic subtype. a) Design dataset (80.6% concordance, 154/191). b) Hold-out dataset (78.9% concordance, 30/38). c) Replication dataset (45.5% concordance, 5/11). d) GEX GSE161501 dataset (100% concordance, 19/19). e) GEX NOPHO GSE228632 dataset (100.0% concordance, 50/50). T-ALL patients (n = 25) were excluded. Subtypes with no patients represented are colored with blue on the y-axis. ALLSorts multiclass predictions are denoted in grey.

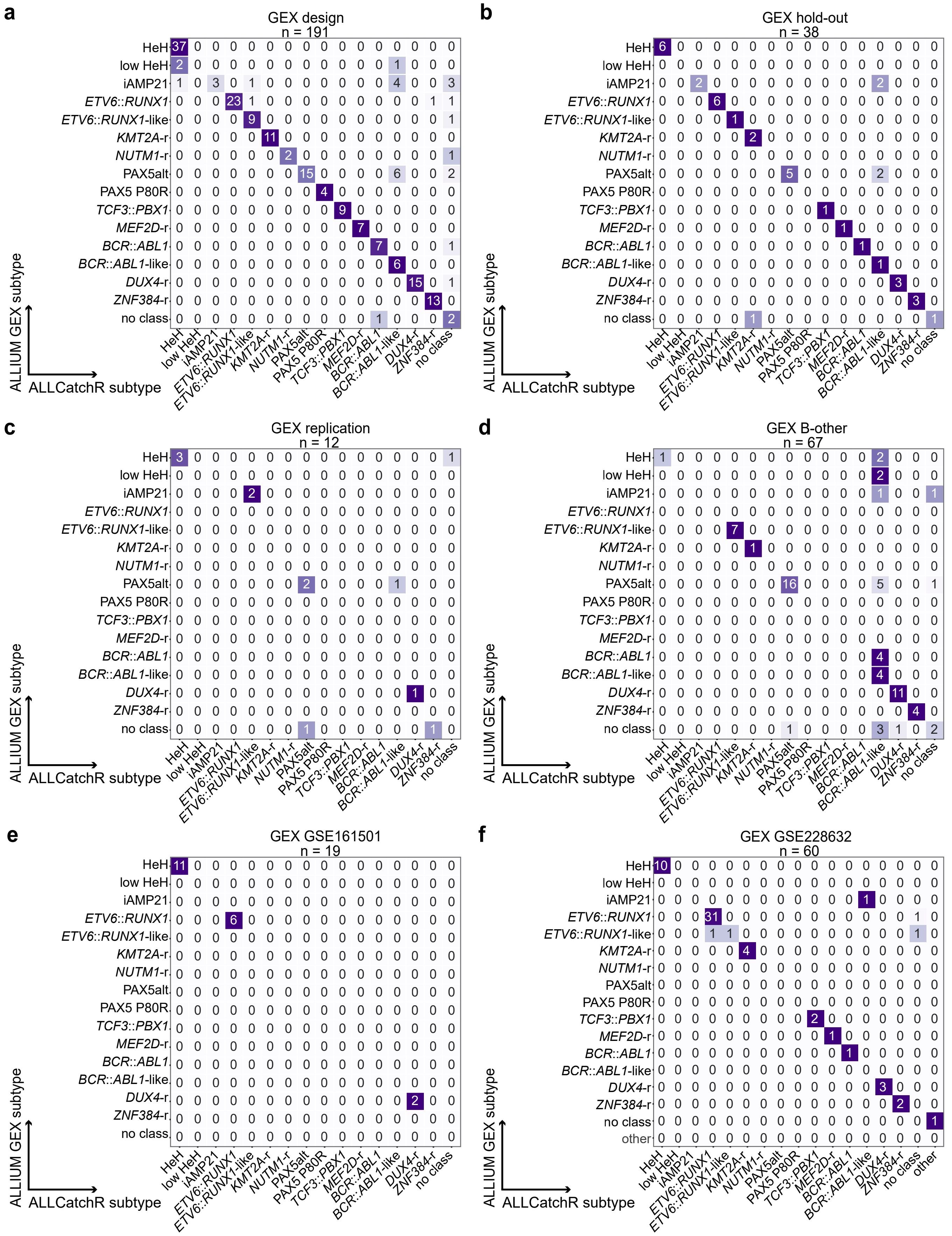

Supplementary Figure 11. Subtype predictions for ALLIUM GEX and ALLCatchR[7]. a) Design dataset 85.3% concordance (163/191). b) Hold-out dataset 86.8% concordance (33/38). c) Replication dataset 50% concordance (6/12). d) Discovery (B-other) dataset 68.7% concordance (46/67). e) GEX GSE161501 100% concordance (19/19), f) GEX NOPHO GSE228632 dataset 91.7 % concordance (55/60). T-ALL patients (n = 25) were excluded.

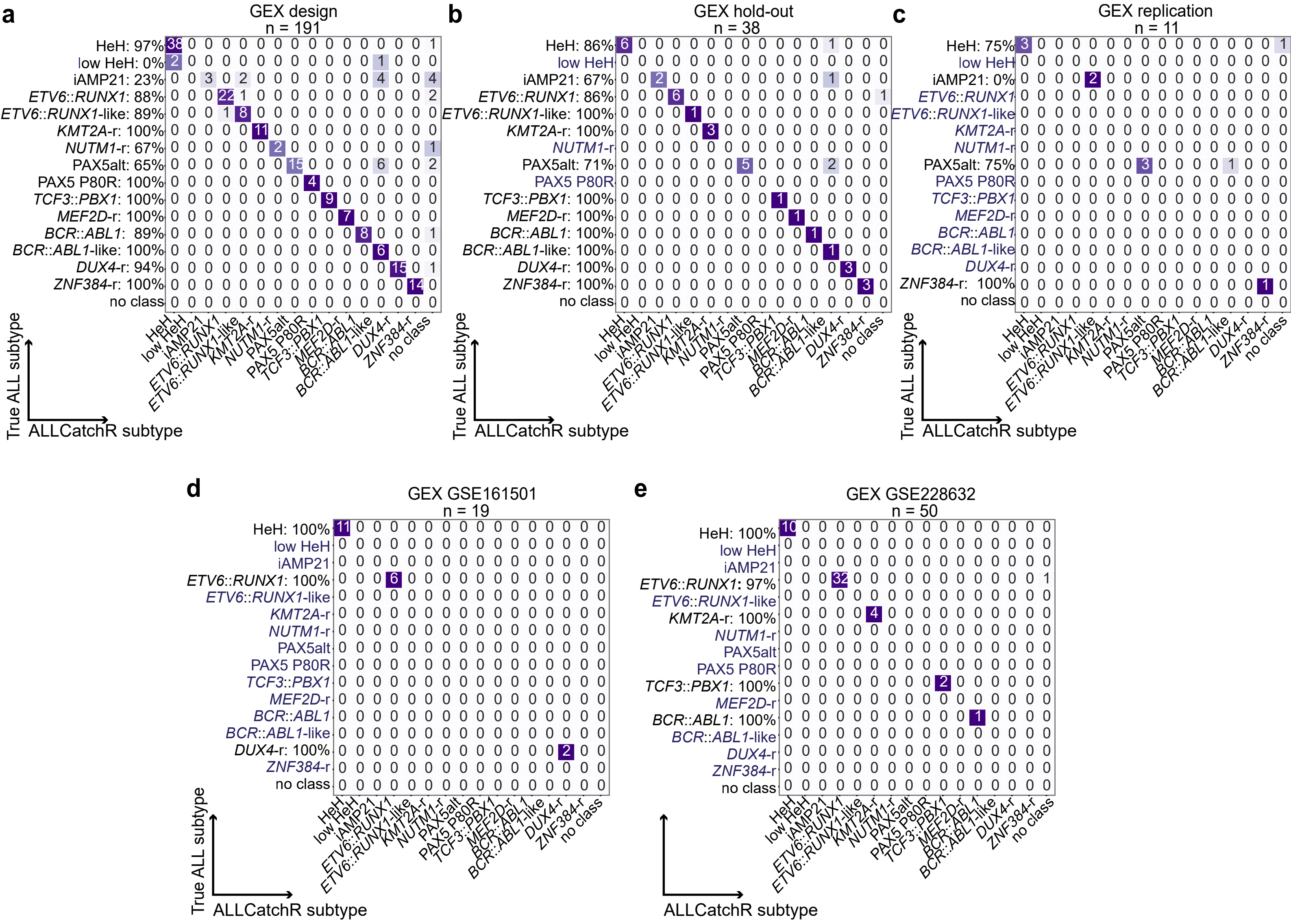

Supplementary Figure 12. Subtype predictions by ALLCatchR[7] vs true cytogenetic subtype. a) Design dataset (84.8% concordance, 162/191). b) Hold-out dataset (86.8% concordance, 33/38). c) Replication dataset (63.6% concordance, 7/11). e) GEX GSE161501 dataset (100%, 19/19). f) GEX NOPHO GSE228632 dataset (98.0%, 49/50). T-ALL patients (n = 25) were excluded. Subtypes with no patients represented are indicated with blue color labels on the y-axis.

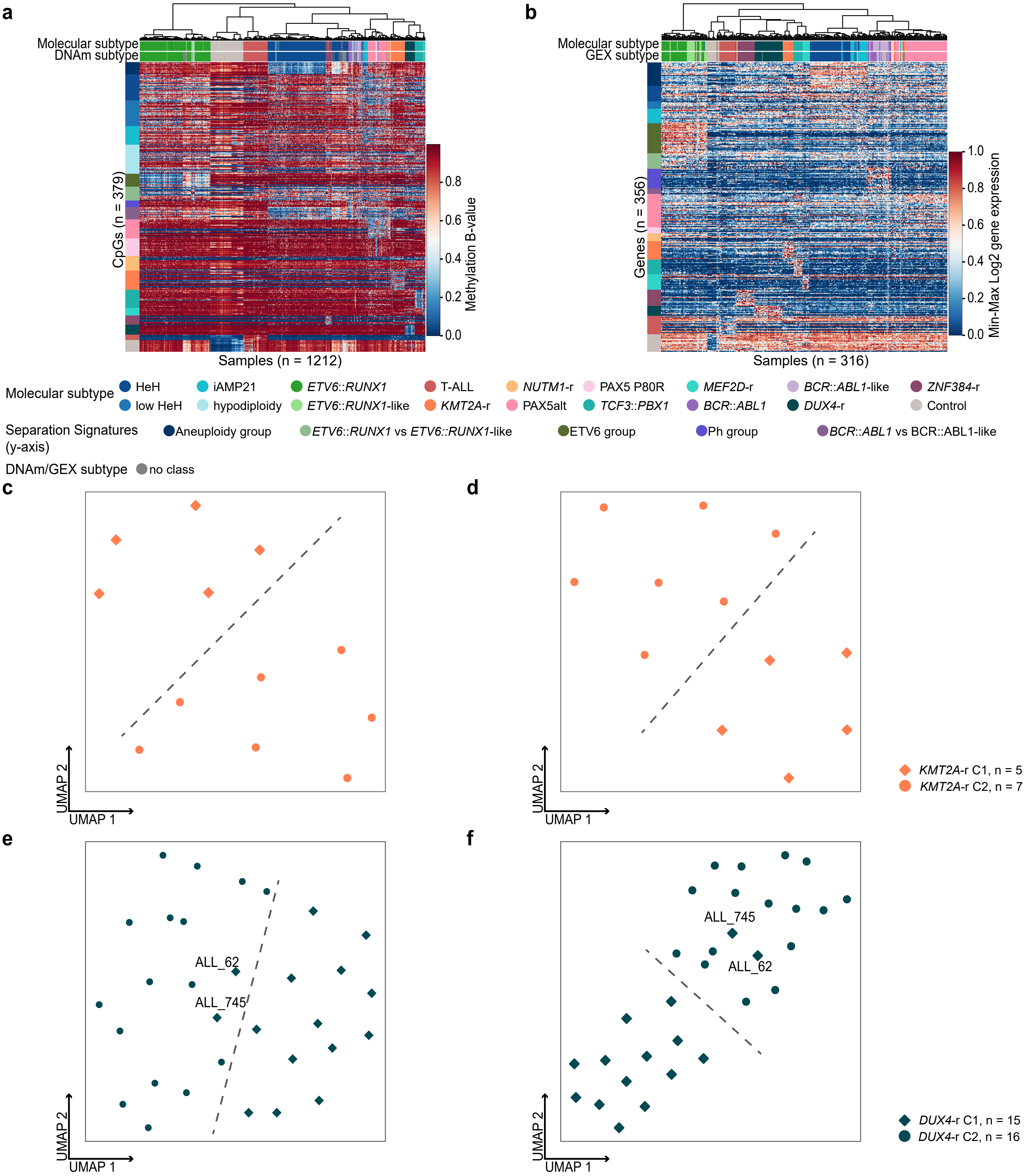

Supplementary Figure 13. Unsupervised visualization after re-classification of B-other. a) Unsupervised hierarchical clustering based on DNAm levels of 379 CpG sites across molecularly defined (n = 971), control (n = 139) and newly re-characterized B-other samples (n = 102). b) Unsupervised hierarchical clustering based on GEX levels of 356 genes across molecularly defined (n = 248), control (n = 12) and newly re-characterized B-other samples (n = 56). c) UMAP plot based on the 356 ALLIUM GEX genes and 12 patients with *KMT2A*-r. Two clusters C1 (n = 5) and C2 (n = 7) are highlighted. d) UMAP plot based on 2,695 genes for distinguishing *KMT2A*-r clusters as determined by Brady et al[8]. The same two clusters are observed in panels c and d. e) UMAP plot based on the 379 ALLIUM genes across 31 *DUX4*-r samples. Two clusters are observed, C1 (n = 15) and C2 (n = 16). f) UMAP plot based on the 2,039 genes used for distinguishing *DUX4*-r clusters as determined by Brady et al[8]. Two outliers from the C1 cluster (ALL_62 and ALL_745) are highlighted in panels e and f.

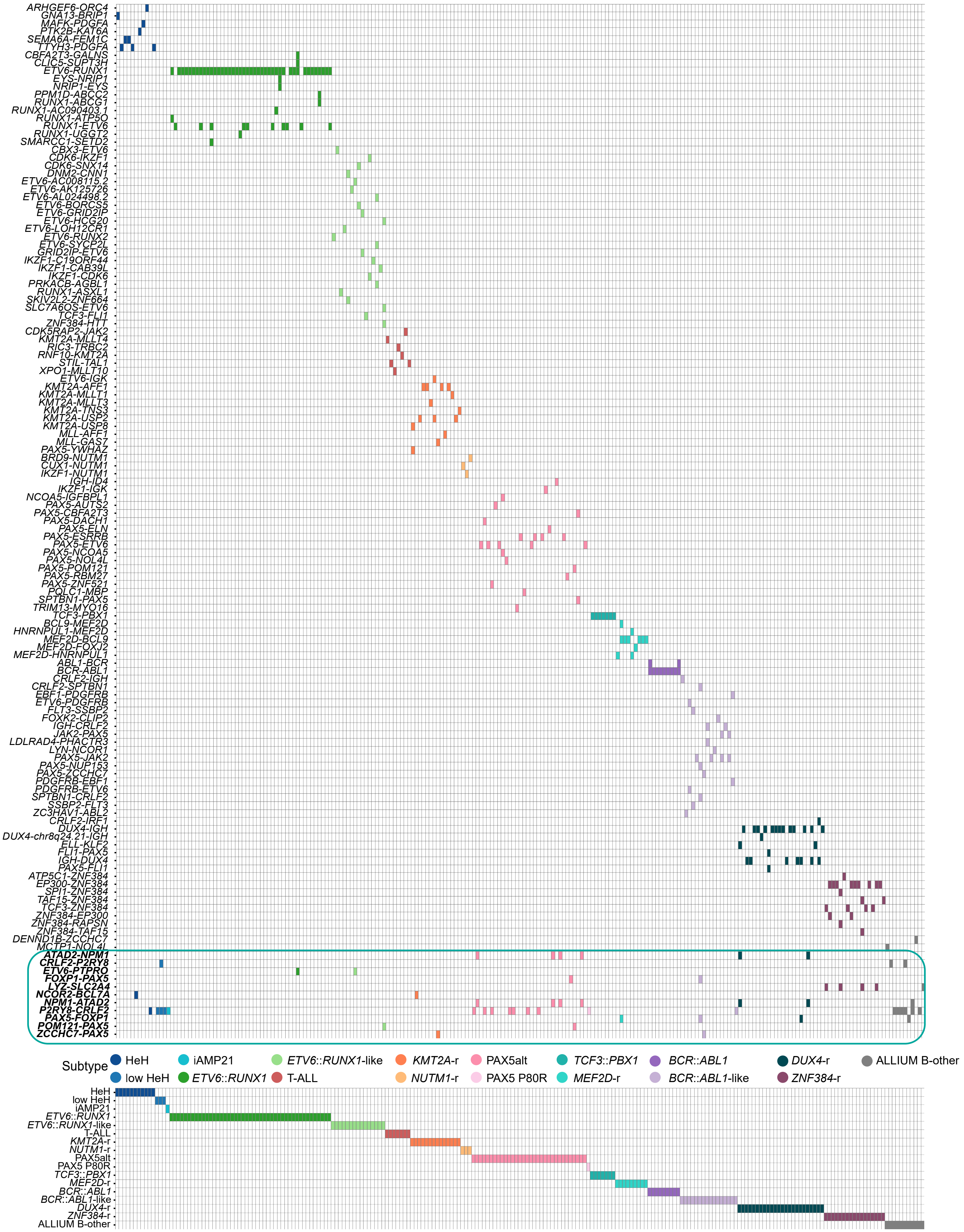

Supplementary Figure 14. Fusion gene distribution across 225 ALL patients after molecular reclassification. The patients are denoted in columns and fusion genes in rows. In total 131 unique fusion genes (including the reciprocal fusion genes) were detected. The eleven fusion genes that appeared across multiple subtypes are denoted within the blue rounded rectangle with bold italics.

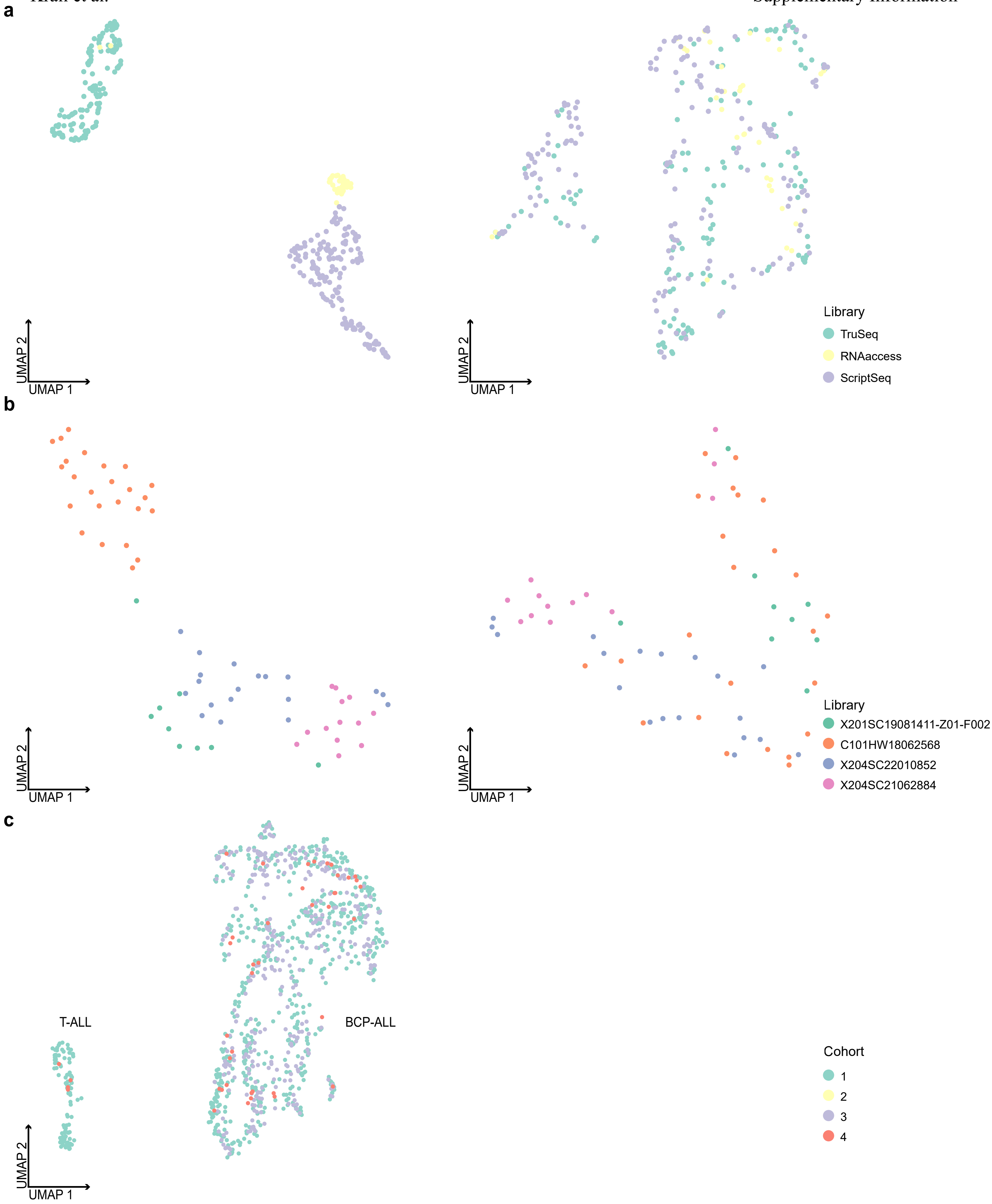

Supplementary Figure 15. Batch effects in the gene expression (GEX) and DNA methylation (DNAm) data. a) Unsupervised dimensionality reduction with UMAP plots visualizing the GEX dataset (n = 328 samples) before (left panel) and after (right panel) batch correction for the three preparation libraries. b) UMAP plots visualizing GEX data (n = 65 samples) from the NOPHO GSE228632 dataset before (left panel) and after (right panel) batch correction for four preparation libraries. c) UMAP plots visualizing the DNAm data (n = 1125 samples) across the four cohorts. The bottom left cluster containing a majority of patients from cohort 1 arises from samples with T-ALL. As there were no large batch effects observed in the DNAm data, batch correction was not performed.

Supplementary References

1. Marincevic-Zuniga, Y. *et al.* Transcriptome sequencing in pediatric acute lymphoblastic leukemia identifies fusion genes associated with distinct DNA methylation profiles. *J. Hematol. Oncol.J Hematol Oncol* **10**, 148 (2017).
2. Lee, S.-T. *et al.* Epigenetic remodeling in B-cell acute lymphoblastic leukemia occurs in two tracks and employs embryonic stem cell-like signatures. *Nucleic Acids Res.* **43**, 2590–2602 (2015).
3. Diedrich, J. D. *et al.* Profiling chromatin accessibility in pediatric acute lymphoblastic leukemia identifies subtype-specific chromatin landscapes and gene regulatory networks. *Leukemia* **35**, 3078–3091 (2021).
4. Tibshirani, R., Hastie, T., Narasimhan, B. & Chu, G. Diagnosis of multiple cancer types by shrunken centroids of gene expression. *Proc. Natl. Acad. Sci.* **99**, 6567–6572 (2002).
5. Sayyab, S. *et al.* Mutational patterns and clonal evolution from diagnosis to relapse in pediatric acute lymphoblastic leukemia. *Sci. Rep.* **11**, 15988 (2021).
6. Schmidt, B. *et al.* ALLSorts: an RNA-Seq subtype classifier for B-cell acute lymphoblastic leukemia. *Blood Adv.* **6**, 4093–4097 (2022).
7. Beder, T. *et al.* The Gene Expression Classifier ALLCatchR Identifies B-cell Precursor ALL Subtypes and Underlying Developmental Trajectories Across Age. *HemaSphere* **7**, e939 (2023).
8. Brady, S. W. *et al.* The genomic landscape of pediatric acute lymphoblastic leukemia. *Nat. Genet.* **54**, 1376–1389 (2022).
